# SLECA: A Single-Cell Atlas of Systemic Lupus Erythematosus Enabling Rare-Cell Discovery Using Graph Transformer

**DOI:** 10.34133/csbj.0163

**Published:** 2026-07-13

**Authors:** Maoteng Duan, Yao Shi, Hao Tian, Qiuqin Wu, Xiaoying Wang, Bingqiang Liu

**Affiliations:** ^1^ School of Mathematics, Shandong University, Jinan, Shandong 250100, China.; ^2^ Shandong National Center for Applied Mathematics, Jinan, Shandong 250100, China.; ^3^ State Key Laboratory of Cryptography and Digital Economy Security, Shandong University, Jinan, Shandong 250100, China.

## Abstract

Systemic lupus erythematosus (SLE) is a complex autoimmune disease with multisystem involvement and marked interpatient heterogeneity. This heterogeneity has hindered precise characterization of the immune dysregulatory mechanisms underlying the disease. While rare immune cell populations are increasingly recognized as critical drivers of disease pathogenesis and progression, the lack of sufficiently powered, comprehensive single-cell transcriptomic resources has limited their systematic identification and characterization. To address this gap, we present SLECA, the first large-scale single-cell RNA sequencing atlas of SLE, together with a novel graph-transformer framework for the interpretable discovery and analysis of disease-relevant rare-cell populations. SLECA integrates 366 samples with standardized clinical and biological metadata, providing an atlas of SLE with a unified analytical framework. Through scalable integration and systematic analysis, SLECA resolves 54 distinct cell types, including rare populations of potential disease relevance. Notably, we identify double-negative T cells (DNTs) as a disease-expanded population whose abundance correlates with clinical severity. In silico perturbation analyses predict shifts in DNT-cell states following perturbation of JUN and EGR1, suggesting potential regulatory roles in DNT-cell transcriptional programs.

## Introduction

Systemic lupus erythematosus (SLE) is a prototypical autoimmune disease that affects approximately 3.4 million individuals worldwide [1]. It is characterized by a breakdown of immune tolerance, persistent autoreactivity, and chronic inflammation, leading to progressive multi-organ damage involving the skin, blood, joints, and brain. In severe cases, renal involvement manifests as lupus nephritis, a major cause of morbidity and mortality [2]. Despite decades of research, the biological basis of SLE remains incompletely understood, largely due to its profound clinical and molecular heterogeneity, which varies across patients and evolves over time, posing major challenges for diagnosis and treatment [3].

Accumulating evidence indicates that this heterogeneity arises primarily from immune dysregulation at the cellular level [4]. In this process, rare immune subsets such as double-negative T cells (DNTs; CD3^+^CD4^−^CD8^−^ T cells) and plasmacytoid dendritic cells (pDCs) have been implicated as key drivers of immune imbalance and disease progression in SLE [5,6]. Although their potential impact on the disease has been preliminarily identified, the specific characteristics, functions, and mechanisms of these rare populations in immune dysregulation remain poorly defined. A major barrier is the lack of sufficiently powered, systematic analyses capable of robustly identifying and characterizing rare-cell states across heterogeneous patient cohorts.

Recent advances in single-cell RNA sequencing (scRNA-seq) have enabled high-resolution characterization of cellular heterogeneity in SLE, including the detection of rare and transitional cell populations and the reconstruction of disease-associated regulatory programs [7,8]. However, existing SLE scRNA-seq datasets remain fragmented across cohorts and platforms, hindering cross-study integration and the construction of a unified atlas. Moreover, standard clustering methods such as Leiden [9] and Louvain [10] are sensitive to parameter settings and often fail to robustly identify rare populations in complex disease settings [11]. Although several specialized rare-cell detection methods, including FIRE [12], GapClust [13], TooManyCells [14], and GiniClust [15], have been proposed, their robustness and scalability remain limited in highly heterogeneous autoimmune diseases. While MarsGT [7] performs well in rare population detection, its broader application is constrained by the scarcity of matched single-cell assay for transposase-accessible chromatin sequencing (scATAC-seq) data, thereby restricting mechanism-level integrative inference [16].

To address these challenges, we present SLECA (systemic lupus erythematosus cell atlas), the first comprehensive scRNA-seq atlas of SLE. SLECA integrates all publicly available SLE scRNA-seq datasets, encompassing 366 samples and over 4 million cells from 8 studies. In addition, SLECA incorporates SarsGT, a novel graph-transformer framework, as its core analytical tool for accurate and interpretable rare-cell discovery. Through a unified analysis pipeline, we identify and annotate 54 distinct cell types, including 3 biologically important rare populations: pDCs, CD8^+^ mucosal-associated invariant T (MAIT) cells, and DNT cells. Notably, DNT cells are expanded in patients with SLE and are comparatively rare in healthy individuals, highlighting their potential as biomarkers of disease activity. Further in silico perturbation analysis suggests that knockout of the key transcription factors EGR1 and JUN was associated with shifts in inferred DNT-cell states toward conventional T cell phenotypes, shedding light on the potential role of these factors in immune regulation. Beyond cell annotation, SLECA provides multiple analytical modules, including gene importance assessment, functional enrichment, cell–cell communication, and regulatory network modeling, thereby establishing a systematic and actionable framework for exploring the immune microenvironment of SLE. By establishing a unified and scalable single-cell atlas, SLECA fills a critical gap in SLE research and provides key data and methodological support for uncovering disease mechanisms, discovering novel biomarkers, and identifying therapeutic targets, thereby offering substantial scientific and translational value.

## Methods

### Data collection

We systematically searched and downloaded publicly available human scRNA-seq datasets related to SLE from the Gene Expression Omnibus (GEO) database. In total, SLECA integrated 366 samples, including 346 samples from 7 SLE-related studies (226 SLE samples and 120 control samples) and an additional control dataset comprising 20 healthy individuals (see Tables S1 to S9 for dataset details and metadata).

### Data preprocessing

Preprocessing was performed using Scanpy v1.9.1 [17] and AnnData v0.8.0 [18]. Low-quality cells and lowly expressed genes were removed, and cells with abnormally high mitochondrial transcript proportions were excluded. Thresholds were set based on a unified baseline and adjusted according to dataset-specific quality-control distributions (sample counts are in Table S11). Doublets were identified and removed using Scrublet v0.2.3 [19] with default settings. Cross-sample integration was performed using scVI v1.1.6 [20], with the preserved raw unique molecular identifier (UMI) counts as input. UMI counts were then normalized on a per-cell basis, log1p-transformed, and used for highly variable gene (HVG) identification, visualization, and other conventional analyses.

### Rare population-friendly clustering with SarsGT

To enable robust identification of rare-cell populations, SLECA incorporates SarsGT, a single-cell clustering framework adapted from our previously developed rare-cell discovery model, MarsGT [7]. Unlike MarsGT, which requires paired multi-omics data, SarsGT operates solely on scRNA-seq data and is therefore suitable for large-scale transcriptomic atlas analysis where matched chromatin accessibility profiles are often unavailable. In addition, SarsGT introduces 3 key modifications: cell-conditioned probabilistic subgraph sampling to better capture rare-cell-associated transcriptional signals, a bidirectional cell–gene heterogeneous graph to preserve mutual gene–cell dependencies, and structure-preserving representation learning to retain the original gene–cell relational structure during embedding. Its core workflow includes heterogeneous graph construction, probabilistic subgraph sampling, representation learning, model training, and cell cluster assignment.

### Heterogeneous graph construction

SarsGT begins with the raw scRNA-seq data, denoted as $X^{R}\in {\mathbb{R}}^{M\times N}$, where M is the number of genes and N is the number of cells. To capture the complex interactions between genes and cells, we construct a gene–cell heterogeneous graph denoted by $G=\left(V,\;E\right)$, where V is the set of nodes and E denotes the edge set. Specifically, $V=V^{G}\cup V^{C}$, where $V^{G}=\left\{v_{i}^{G}|i=1,2,\cdots ,M\right\}$ denotes the set of gene nodes and $V^{C}=\left\{v_{k}^{C}|k=1,2,\cdots ,N\right\}$ denotes the set of cell nodes. The edges E in the graph are established between gene and cell nodes when $X_{\mathrm{ik}}^{R}>0$, indicating a nonzero expression value for the gene in the respective cell. The initial feature representation for the nodes is derived from the raw data, denoted as $F=F^{C}\cup F^{G}$, where $F^{C}={\left(X^{R}\right)}^{T}$ represents the feature matrix for cells, and $F^{G}=X^{R}$ represents the feature matrix for genes.

### Subgraph sampling

To enable efficient model training on large-scale datasets and enhance the detection of rare-cell populations, SarsGT employs a subgraph sampling strategy before model training. This approach is motivated by the biological insight that genes with high expression in a small subset of cells play a pivotal role in both cell clustering and the identification of rare cell types. To construct the subgraphs for model training, SarsGT calculates a sampling probability for each gene–cell pair $\left(i,\;k\right)$ as follows:

Step 1: For each gene i and cell k, an initial importance score $S_{\mathrm{ik}}$ is calculated. This score is defined as the ratio of the gene’s expression in the target cell k relative to its total expression across all cells:

$$S_{\mathrm{ik}}=\frac{X_{\mathrm{ik}}^{R}}{\sum_{c}X_{\mathrm{ic}}^{R}}$$ (1)

Step 2: The final sampling probability $P\left(v_{i}^{G},v_{k}^{C}\right)$ is derived through a conditional normalization process. For a given cell $k_{0}$, genes whose expression exceeds a threshold β (defined as the first quartile of the gene expression value in $k_{0}$) are selected, forming a high-expression gene subset $I_{k_{0}}=\left\{i|X_{ik_{0}}^{R}>\beta \right\}$. The sampling probability for gene $i_{0}$ in cell $k_{0}$ is then calculated as the ratio of its importance score $S_{i_{0}k_{0}}$ to the sum of importance scores for all genes in the subset $I_{k_{0}}$:

$$P\left(v_{i_{0}}^{G},\;v_{k_{0}}^{C}\right)=\frac{S_{i_{0}k_{0}}}{\sum_{i\in I_{k_{0}}}S_{ik_{0}}}$$ (2)

Step 3: Based on the above probability, each training subgraph is constructed by randomly sampling 30 cells and sampling the associated gene nodes (i.e., neighbors) for each cell according to the computed probability distribution. To manage computational complexity, the number of neighboring nodes for each cell is limited to the smaller value between the total number of genes $N_{G}$ and 20, i.e., $\min\left(N_{G},\;20\right)$, ensuring that the subgraph size remains manageable.

### Feature learning and updating

The SarsGT model is trained on sampled subgraphs, using a stack of L heterogeneous graph transformer (HGT) layers to iteratively update node features. We denote the feature representation of node v at layer las $h_{v}^{l}$. For the nodes $V^{G}$ and $V^{C}$, their initial features $h_{V_{G}}^{0}$ and $h_{V_{C}}^{0}$ are directly derived from the initial feature matrices $F^{G}$ and $F^{C}$, respectively. To project different node types into the same latent space, we apply type-specific linear transformations W to the node features:

$$h_{v}^{l}=W_{\phi \left(v\right)}\cdot h_{v}^{l-1}$$ (3)

Here, $\phi \left(v\right)$ represents the type of node v (either gene or cell), and $W_{\phi \left(v\right)}$ is the corresponding learnable weight matrix.

Next, a multi-head attention mechanism is applied by dividing $h_{v}^{l}$ into h heads. For the head h at layer l, linear mapping functions are used: query (Q), key (K), and value (V). For each node v, the transformed representations are:

$$Q^{h}\left(v\right)=W_{Q}^{h}\cdot h_{v}^{l}$$ (4)

$$K^{h}\left(v\right)=W_{K}^{h}\cdot h_{v}^{l}$$ (5)

$$V^{h}\left(v\right)=W_{V}^{h}\cdot h_{v}^{l}$$ (6)

To compute the attention between node v and its neighbors $N\left(v\right)$ in head h, we define an attention function estimating the importance of neighboring node $v_{n}$:

$$I_{v_{n}}=K^{h}\left(v_{n}\right)W_{\theta \left(v_{n},\;v\right)}^{\mathrm{att}}Q^{h}{\left(v\right)}^{T}$$ (7)

where $W_{\theta \left(v_{n},v\right)}^{\mathrm{att}}$ is a transformation matrix capturing edge type-specific features, and $\theta \left(\cdot \right)$ denotes the edge type. The overall multi-head attention is then computed as:

$$\begin{array}{c} \mathrm{att}\left(v_{n},v\right)=\underset{\forall v_{n}\in N\left(v\right)}{\text{Softmax}}\left(\underset{h}{\|}\left({\mathrm{ATT}}_{\text{head}}^{h}\left(v_{n},\;v\right)\right)\right) \end{array}$$ (8)

$${\mathrm{ATT}}_{\text{head}}^{h}\left(v_{n},\;v\right)=I_{v_{n}}\cdot \frac{\mu \langle v_{n},\;v\rangle }{\sqrt{d}}$$ (9)

where $\|\left(\cdot \right)$ denotes the concatenation function, ${\mathrm{ATT}}_{\text{head}}^{h}\left(v_{n},\;v\right)$ represents the attention weight of the hth head, and μ is a prior scaling function that accounts for the meta-relation characteristics between nodes and edges. Finally, the attention coefficients are normalized through the Softmax function to ensure that the attention weights between node pairs satisfy a valid probability distribution.

The information of node $v_{n}$ in head h can be transmitted to node v through:

$$\mathrm{mes}\left(v_{n},v\right)=\underset{h}{\|}V^{h}\left(v_{n}\right)W_{\theta \left(v_{n},v\right)}^{\mathrm{MSG}}$$ (10)

where $W_{\theta \left(v_{n},v\right)}^{\mathrm{MSG}}$ is a transformation matrix similar to $W_{t\left(v_{n},v\right)}^{\mathrm{att}}$.

To update the feature of node v, the final step at layer l aggregates the information obtained from its neighboring nodes $N\left(v\right)$, based on $h_{v}^{l-1}$ and the intermediate representation $h_{v}^{l^{\prime }}$:

$$\begin{array}{c} h_{v}^{l^{\prime }}=\underset{\forall v_{n}\in N\left(v\right)}{\text{Aggregate}}\left(\mathrm{att}\left(v_{n},v\right)\cdot \mathrm{mes}\left(v_{n},v\right)\right) \end{array}$$ (11)

$$h_{v}^{l}=\omega \left(\text{ReLU}\left(h_{v}^{l^{\prime }}\right)\right)+\left(1-\omega \right)h_{v}^{l-1}$$ (12)

where ω is a trainable parameter and ReLU is the activation function. The aggregation function Aggregate can be implemented using average pooling, max pooling, or other pooling operations. The final embedding of the target node $v_{}$ is obtained by stacking the information from all L HGT layers. In the last HGT layer, the attention score between gene $i_{0}\;$and cell $k_{0}$ is defined as:

$$\begin{array}{c} {\text{Score}}_{\mathrm{att}}\left(i_{0},\;k_{0}\right)=\sqrt{\sum_{h}{\left({\mathrm{ATT}}_{\text{head}}^{h}\left(i_{0},\;k_{0}\right)\right)}^{2}} \end{array}$$ (13)

This score reflects the relative importance of gene $i_{0}$ to cell $k_{0}$. The formulation is designed to balance the contributions of positive and negative attention scores to the target node, ensuring that the model can fairly attend to information from different directions, thereby capturing the complex relationships between nodes more comprehensively. After multiple layers of updates, the final feature of node v is obtained.

### Model optimization and training

After the embeddings are calculated, the genes and cells obtain updated embeddings, denoted as $\left\{h_{V}^{l}|V=V^{C}\cup V^{G}\right\}$. The updated embeddings of cells $h_{V^{C}}^{l}$ are denoted as P after normalizing by column sums. Each row of matrix P represents a cell, each column corresponds to a manually defined reduced-dimensional set, and each element indicates the probability that a cell belongs to a specific cluster. In SarsGT, subgraph training is conducted in an unsupervised manner, with the loss function defined as follows: (a) Kullback–Leibler (KL) divergence loss, which preserves the structural consistency of the data. (b) Cosine similarity loss, which ensures that the features of cells within the same type are sufficiently similar. (c) Regularization loss, which promotes smoother optimization and faster convergence. The overall training objective is defined as:

$${\text{Loss}}_{\text{train}}={\mathrm{KL}}_{\text{loss}}+{\mathrm{Cos}}_{\text{loss}}+{\mathrm{Reg}}_{\text{loss}}$$ (14)

The KL divergence loss is formulated as:

$${\mathrm{KL}}_{\text{loss}}=\mathrm{KL}\left(h_{V^{G}}^{l}\cdot {\left(h_{V^{C}}^{l}\right)}^{T},\;X^{R}\right)$$ (15)

The cosine similarity loss is defined as:

$${\mathrm{Cos}}_{\text{loss}}=-\sum_{C}\text{Cosine}\left(\left\{h_{V^{C}}^{l}|C\in \text{same cluster}\right\}\right)$$ (16)

The regularization loss is defined as:

$${\mathrm{Reg}}_{\text{loss}}={\text{Smoothing}}_{\text{cross}}\left(P,\;L,\;\varepsilon \right)$$ (17)

Here, $L=\left\{l_{1},\;l_{2},\ldots ,\;l_{N}\right\}$ represents the cluster labels obtained from Louvain clustering on the scRNA-seq data, and ε is a smoothing factor.

The smoothing cross-entropy loss is defined as:

$$\begin{array}{c} {\text{Smoothing}}_{\text{corss}}\left(P,\;L,\;\varepsilon \right)=-\sum_{k=1}^{N}\sum_{l=1}^{l_{N}}y_{\mathrm{kl}}*\text{log }p_{\mathrm{kl}} \end{array}$$ (18)

ykl=1−lN−1lN∗εiflk=lεlNiflk≠l (19)

where $p_{\mathrm{kl}}$ represents the predicted probability of the given cell k belonging to the class l. These training objectives collectively help the model make confident predictions during optimization while maintaining stability, enabling SarsGT to learn robust cell-type representations across subgraphs.

### Cell cluster assignment and cell-type annotation

For each sample, the model output included a posterior probability matrix of cell clusters for each cell and cell-level gene attention scores. Cells were assigned to clusters based on the maximum posterior probability, and cluster identities were annotated using canonical marker genes (Table N2). The results were visualized using dot plots, violin plots, and Uniform Manifold Approximation and Projection (UMAP) plots.

### Differential expression and functional enrichment

Differential expression analysis was performed in Scanpy using the Wilcoxon rank-sum test, comparing each target cluster with all remaining cells. Gene set enrichment analysis was conducted using GSEApy [21] via the Enrichr Application Programming Interface (API) [22], primarily referencing the Kyoto Encyclopedia of Genes and Genomes (KEGG) database [23].

### DNT-cell visualization and groupwise statistical analysis

To compare the distribution of DNT/T cell ratios between the SLE and non-SLE groups, we plotted histograms with kernel density estimates (KDEs) using Seaborn and Matplotlib. Ratios were binned at 0.1 intervals from 0 to 1. Group-wise differences between samples with and without detectable DNT cells were evaluated using a one-sided Mann–Whitney *U* test, with the prespecified alternative hypothesis that samples with detectable DNT cells would have higher systemic lupus erythematosus disease activity index (SLEDAI) scores.

### Statistical modeling and regression analyses

To assess the association between attention-derived molecular features and disease activity, we performed sample-level multivariable linear regression using the statsmodels Python package. For each sample with available SLEDAI information, the SarsGT-derived attention scores of the top 10 selected genes were used as predictors, with SLEDAI score as the dependent variable. An ordinary least squares (OLS) model was fitted, assuming linear relationships between predictors and outcome, independence of observations across samples, homoscedastic residuals, and approximately normally distributed error terms. The fitted regression coefficients were used to compute a model-based weighted composite score for each sample as the weighted sum of the 10 sample-level attention-derived gene features.

### Inference of cell–cell communication networks

We used CellChat v2 [24] to infer and compare ligand–receptor networks between the SLE and control groups. Differential edges and pathways were assessed using permutation testing followed by false discovery rate (FDR) correction.

### In silico gene perturbation using CellOracle

We used CellOracle v0.20.0 [25], a framework for transcription factor perturbation and cell-state transition modeling, to reconstruct the gene regulatory network (GRN) of DNT cells and perform in silico perturbation simulations.

### Pseudotime trajectory reconstruction and identification of switch genes

Pseudotime trajectories were reconstructed using Monocle v2.26.0 [26] with the DDRTree algorithm. Switch genes were identified using GeneSwitches v0.1.0 [27], which detects genes exhibiting on/off transitions along the pseudotime axis.

### Transcription factor activity inference and GRN reconstruction

SCENIC v1.1.2.1 [28], which is used to infer regulon activity associated with transcription factors, was applied to the normalized expression matrix of the selected cell populations. Genes with low expression were first removed using the SCENIC gene-filtering procedure. GRNs were then inferred using the GENIE3 algorithm based on transcription factor–target gene coexpression relationships. Coexpression modules were refined through motif enrichment analysis using the human cisTarget database (500-base pair upstream regulatory region database), and regulons were defined as transcription factors together with their predicted target genes supported by motif evidence. Regulon activity in individual cells was quantified using AUCell enrichment scores, which were subsequently used to compare transcription factor activity across cell populations and disease conditions. We also clarified that the downstream analyses were based on continuous AUCell scores rather than binarized regulon activity values.

## Results

### Overview of SLECA

**Fig. 1.**
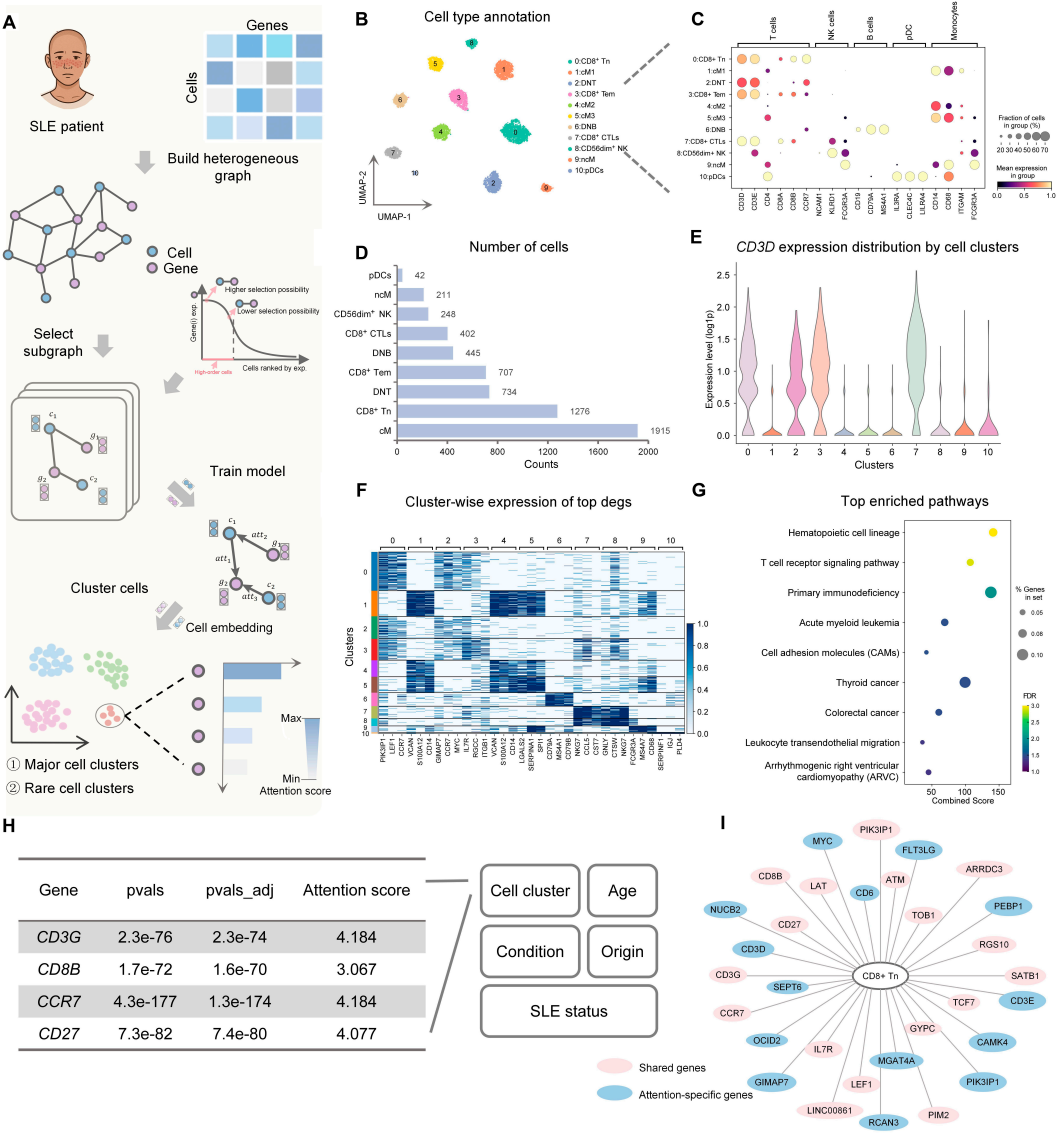
Overview of SLECA. (A) Schematic overview of the SarsGT framework for scRNA-seq analysis. (B to I) Representative SarsGT outputs generated from the cSLE4 (JB17004) sample in GSE135779. (B and C) UMAP plot and dot plot of cell-type annotations. (D) Bar plot of cell counts across cell types. (E) Violin plots showing gene expression across cell types. (F) Heatmap of DEGs among cell types. (G) Functional enrichment analysis of DEG. (H) Gene attention scores across different cell types inferred by SarsGT. (I) Network visualization of genes associated with CD8^+^ Tn cells identified by the attention-based approach. Pink nodes represent genes shared with DEG analysis, whereas blue nodes are specific to the attention mechanism.

SLECA is designed to address the challenges of unstable rare-cell identification and the difficulty in locating cell type-specific key genes within a shared state space in cross-cohort integration. SLECA incorporates SarsGT, a graph-transformer framework designed for interpretable rare-cell discovery. SarsGT models scRNA-seq data as a cell–gene heterogeneous graph and jointly outputs posterior probabilities for cell clusters and cell-level gene attention scores. This architecture enhances the visibility of low-abundance cellular signals while providing a biologically consistent and interpretable measure of gene importance aligned with cell-state variation (see Fig. 1A; Methods).

By leveraging SarsGT, SLECA performs a unified analysis across all samples, yielding multi-layered results. To illustrate the outputs generated by SarsGT using real data, Fig. 1B to I illustrates representative outputs generated by SarsGT from the cSLE4 [JB17004] sample, including cell clustering, marker visualization, gene prioritization, and downstream enrichment analysis. Within a shared embedding space, key immune subsets are resolved, including CD8^+^ naive T cells (CD8^+^ Tn), DNT cells, pDCs, and CD56^dim^ natural killer (NK) cells, thereby enabling cross-cohort comparisons (Fig. 1B and C). The marker genes used for cell-type annotation are listed in Table N2. Meanwhile, cluster compositions are quantified, and marker/representative genes are visualized, providing quantitative descriptions of compositional differences across conditions and populations (Fig. 1D and E). Examples of violin plots for additional genes are shown in Fig. S1. Moreover, SLECA includes differentially expressed genes and pathway enrichment results, for example, when comparing CD8^+^ Tn cells with other subsets, immune-related pathways such as T cell receptor (TCR) signaling are significantly enriched (Fig. 1F and G). In addition, the attention mechanism of SarsGT assigns importance scores to each gene in each SLECA cell and enables comparisons across cell clusters, age groups, disease states, tissue sources, and activity levels (Fig. 1H). In comparison with traditional differentially expressed gene (DEG) analysis (Fig. 1I), attention-based prioritization recovered canonical cell identity markers such as *CD3D* and *CD3E* and prioritized broadly cell-state-associated genes such as *MYC*. These results suggest that SarsGT attention scores provide an additional model-driven perspective for interpreting the cell identity and state representations learned by the model.

To further support the utility of SarsGT for rare-cell discovery, we benchmarked it against commonly used and rare-cell-oriented methods, including Leiden, Louvain, scVI+Leiden, scVI+Louvain, GiniClust, TooManyCells, GapClust, and scGNN2.0 [29]. Across 5 annotated public scRNA-seq datasets covering peripheral blood mononuclear cell (PBMC), liver, lung, and pancreas contexts, SarsGT achieved competitive overall clustering performance based on adjusted Rand index (ARI) and normalized mutual information (NMI) while showing a stronger advantage in rare-cell recovery based on rare-cell F1 score. In particular, SarsGT achieved an overall rare-cell F1 score of 0.78, outperforming the compared methods, while maintaining overall ARI and NMI values of 0.71 and 0.83, respectively (Fig. S2 and Table N1). Ablation analyses further showed that the attention mechanism, heterogeneous graph structure, and subgraph sampling strategy each contributed to rare-cell detection performance (Fig. S3).

### Cross cohort data integration in SLECA

**Fig. 2.**
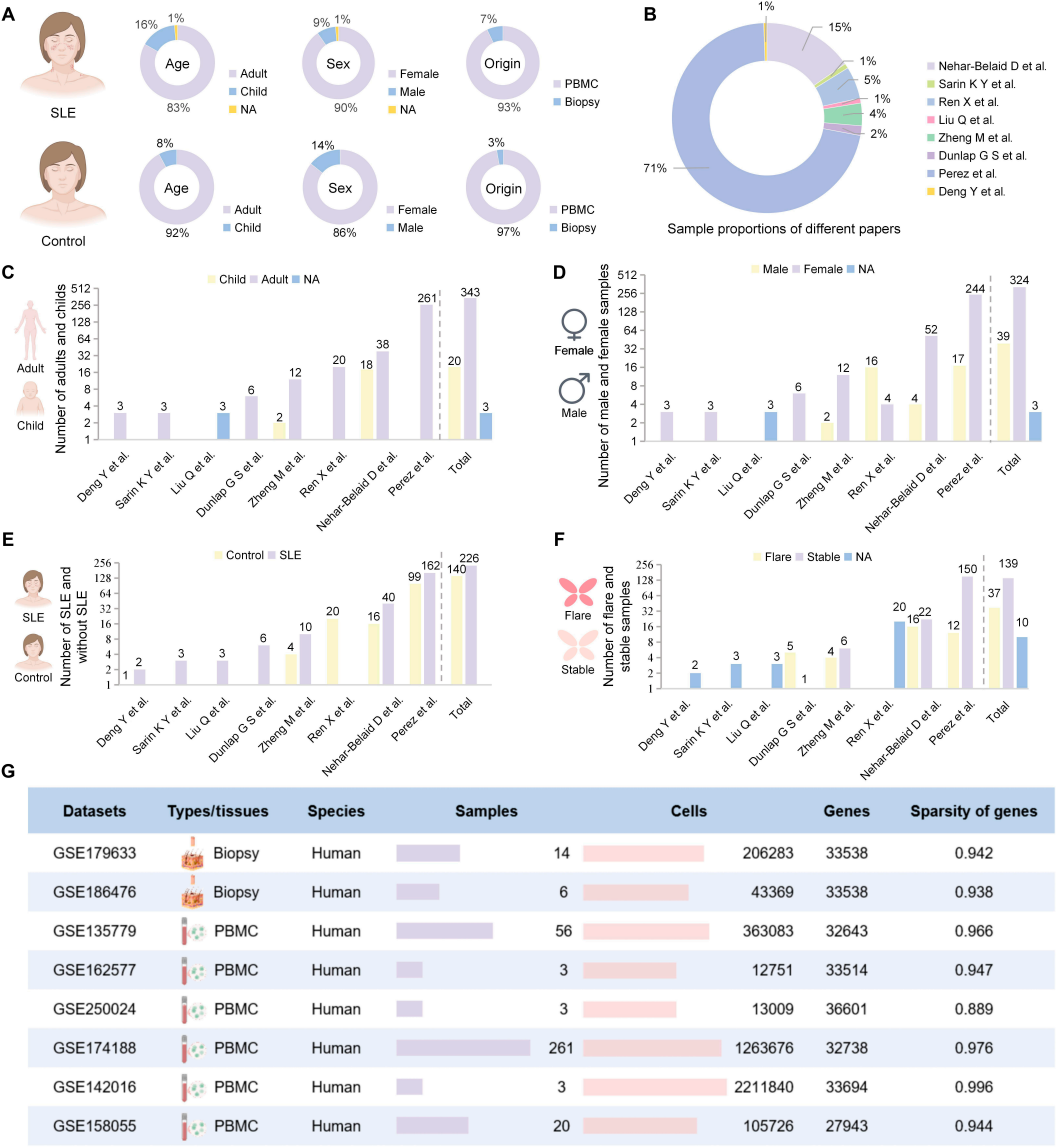
SLECA data characteristics and statistics. (A) Proportions of samples by age, sex, and tissue source in with SLE and without SLE groups. (B) Sample contributions across individual studies. (C to F) Numbers and distributions of samples across studies stratified by (C) age, (D) sex, (E) disease status (SLE versus control), and (F) disease activity (flare vs stable). (G) Dataset-level summary (GEO accession, tissue/source, species, numbers of samples/cells/genes, and gene sparsity).

SLECA compiles a total of 366 human scRNA-seq samples, derived from 7 published SLE studies and 1 healthy control study (all obtained from the GEO database; sample details are provided in Tables S1 to S9). For each sample, we standardized key metadata including age, sex, and tissue of origin (Fig. 2A).

Cross-study integration revealed that most samples originated from large-scale datasets, particularly Perez et al. [30], with the remaining studies serving as supplementary sources (Fig. 2B). When stratified by age and sex (Fig. 2C and D), the cohort was dominated by adult and female donors, consistent with the well-documented epidemiological pattern of SLE, which predominantly affects women of reproductive age. The atlas also includes both with SLE and without SLE samples (Fig. 2E) and records disease activity states (flare/stable; Fig. 2F), enabling case–control and activity-based comparative analyses. Furthermore, for each dataset, we systematically summarized accession numbers, tissue sources, and data characteristics, including sample, cell, and gene counts as well as data sparsity (Fig. 2G), to facilitate methodological reproducibility and cross-cohort benchmarking. Detailed proportions and study contributions are provided in the figure legend and Tables S10 and S11.

### Cell-type composition and distribution across conditions in SLECA

**Fig. 3.**
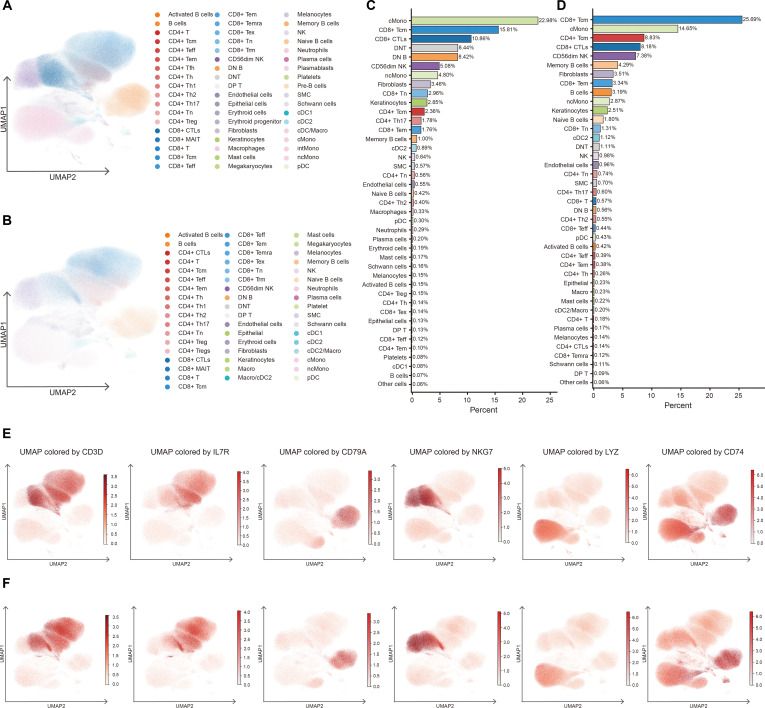
Comprehensive cell-type landscape and marker gene expression in SLECA. (A and B) UMAP visualizations of cell-type distribution in SLE and control groups. (A) SLE group. (B) Control group. (C and D) Bar charts showing the relative proportions of cell types in SLE and control groups. (C) SLE group and (D) control group, highlighting variations in immune cell distribution (e.g., T cells, B cells, and NK cells). (E and F) UMAP visualizations of marker gene expression for major cell types (T cells, B cells, and NK cells) in SLE and control groups. (E) SLE group. (F) Control group.

While cohort-level integration establishes the scope and representativeness of SLECA, its primary value lies in resolving disease-associated heterogeneity at the cellular level. Through SLECA, we annotated and constructed a comprehensive cell-type landscape for both the SLE and control datasets, encompassing over 4 million cells and 54 distinct cell types. UMAP visualizations provided a qualitative overview of the integrated cellular landscape and displayed the annotated cell types in a low-dimensional space (Fig. 3A and B).

To describe differences in cell composition across conditions, we summarized cell-type proportions at the sample level and visualized their relative distributions in bar charts (Fig. 3C and D). These analyses showed differences in the relative abundance of major immune cell populations, including T cells, B cells, and NK cells, between the SLE and control groups. Detailed proportions of other cell types are provided in Tables N3 and N4.

Additionally, we visualized the marker gene expression of major cell types, including T cells, B cells, and NK cells, in both the SLE and control groups. These visualizations revealed gene expression differences between the SLE and control groups (Fig. 3E and F). Other UMAP visualizations of marker gene expression are shown in Fig. S4. These multidimensional analyses of cell types and gene expression not only provide a detailed cell composition map of the SLE immune microenvironment but also lay the foundation for further studies of rare-cell populations in SLE.

### Profiling DNT cells in SLECA for cross cohort expansion and functional characterization

DNTs have drawn increasing attention in SLE due to their potential roles in pathogenic processes. These cells lack both CD4 and CD8 surface markers while expressing αβ or γδ TCRs, and are extremely rare in healthy individuals but markedly expanded in SLE patients [31]. To investigate the role of DNTs in SLE, we focused on this cell population, which has been associated with immune dysregulation and disease severity [6,32].

**Fig. 4.**
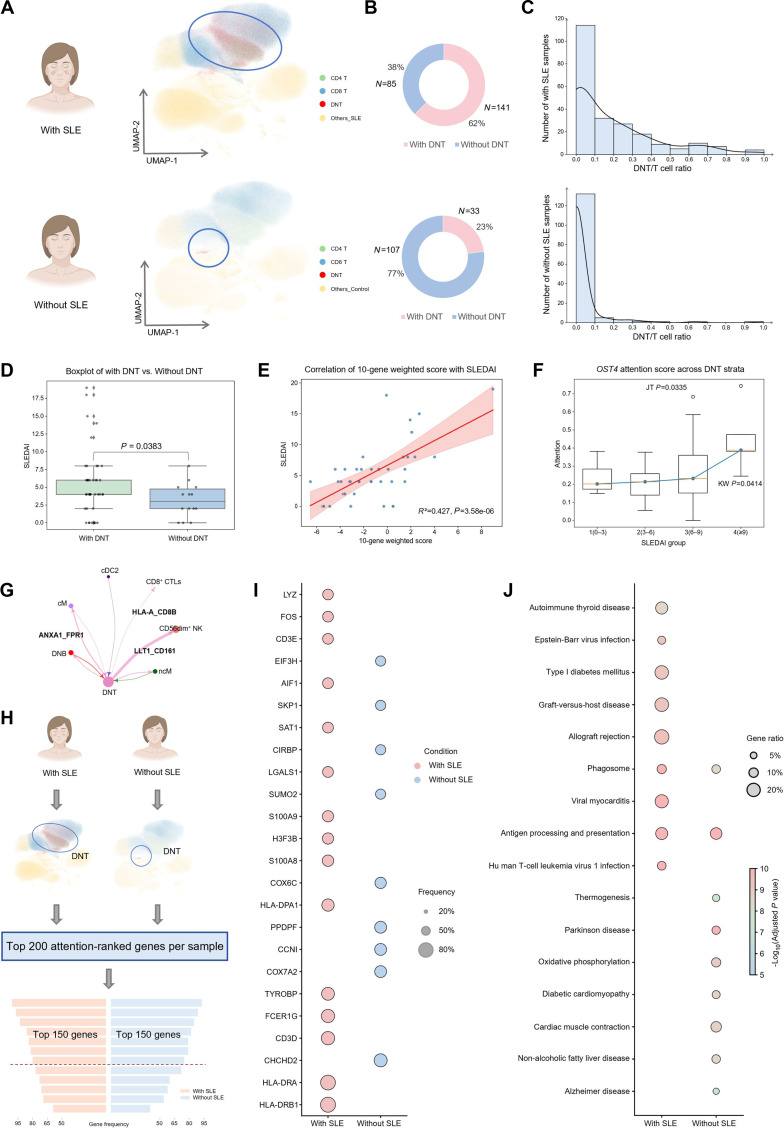
Functional characterization of DNT cells in SLE. (A) UMAP visualization of cellular landscapes across conditions, with DNT cells highlighted in red, CD4/CD8 T cells in green/blue, and other cell types in yellow. (B) Proportion of samples with or without detectable DNT cells in SLE and control groups. (C) Distribution of the proportion of DNT cells among total T cells across samples, shown as a histogram with kernel density estimation (KDE). (D) Comparison of SLEDAI scores between SLE samples with and without DNT cells, assessed using a donor-level one-sided Mann–Whitney *U* test ($P=0.0383;n_{1}=41,n_{2}=14$). Note: the one-sided test in (D) was applied based on the clear directional prior hypothesis derived from the established pathogenic and pro-inflammatory profile of DNT cells. (E) Association between the model-based 10-gene weighted composite score and SLEDAI disease activity score. Each point represents one sample with available SLEDAI information (*n* = 44). The composite score was calculated as the weighted sum of the 10 sample-level SarsGT-derived attention scores of the selected genes, using the corresponding coefficients from the fitted multivariable linear regression model. The red line indicates the fitted linear regression, and the shaded area represents the 95% confidence interval. (F) *OST4* attention scores across 4 ordered SLEDAI strata (0 to 3, 3 to 6, 6 to 9, ≥9), overall differences among groups were assessed using the Kruskal–Wallis test (P=0.0414), and monotonic trends were evaluated using the Jonckheere–Terpstra trend test (P=0.0335). (G) Differential cell–cell communication networks between SLE and without SLE groups. (H) Workflow illustrates the selection of the top 150 key genes based on attention scores. The diagram outlines the stepwise procedure applied to DNT cells from both SLE and control groups, including gene ranking, frequency assessment, and final set determination. (I) Bubble plot showing the distribution of SLE- and control-specific genes in DNT cells, together with their occurrence frequencies. (J) Functional enrichment of DNT-specific genes in SLE.

To characterize DNT-cell alterations in SLE, we examined their distribution and donor-level abundance in SLE and control samples. Group-specific UMAPs provided a qualitative visualization of DNT-cell distributions across groups (Fig. 4A), while quantitative comparisons were performed at the donor level to ensure independence of observations. DNT cells were detected in 62% of SLE donors compared with only 23% of controls (Fig. 4B). Moreover, the fraction of DNT cells among total T cells was shifted toward higher values in SLE (Fig. 4C), supporting donor-level DNT-cell enrichment in SLE. To assess whether this enrichment was influenced by tissue source or unmatched external healthy controls, we performed a PBMC-only sensitivity analysis excluding the skin biopsy datasets GSE179633 and GSE186476, as well as the external healthy control dataset GSE158055. In this restricted analysis, DNT cells remained more frequently detected in SLE than in healthy controls, and the DNT/T-cell ratio also remained higher in SLE (Fig. S5), supporting the robustness of DNT-cell enrichment in SLE. We then evaluated whether this donor-level DNT-cell enrichment was related to clinical disease activity. Donors with detectable DNT cells exhibited significantly higher SLEDAI scores [33] than those without detectable DNT cells (Fig. 4D), supporting an association between donor-level DNT-cell enrichment and higher disease activity [34].

In addition, given that DNT cells may exhibit distinct molecular states across different levels of disease activity, we further analyzed genes prioritized by the SarsGT attention mechanism to explore molecular features associated with DNT-related cell-state variation. A multivariable model incorporating the top 10 attention-derived gene features showed a significant overall association with SLEDAI. The derived model-based 10-gene weighted score was positively correlated with SLEDAI ($R^{2}$ = 0.427, P = 3.58 × 10^−6^; Fig. 4E), suggesting that these model-prioritized DNT-associated molecular features were jointly associated with higher disease activity. Attention-derived gene feature–SLEDAI associations are provided in Fig. S6. We then examined selected high-ranking genes as exploratory examples, including both genes from the top 10 attention-ranked set and several near-top-ranked candidates. Among these, *OST4* (ranked 12th) exhibited a positive trend in attention scores across increasing SLEDAI categories (Fig. 4F), while additional examples, including *ARPC3* (ranked 14th), *MYL6* (ranked 5th), and *SRP14* (ranked 19th), are shown in Fig. S7.

We next investigated whether DNT cells may contribute to the altered immune environment observed in SLE by examining intercellular communication patterns. Differential communication analysis revealed increased predicted interactions between DNT cells and multiple immune cell populations in SLE (Fig. 4G), suggesting broader involvement of DNTs in the immune communication network. Among these interactions, LLT1–CD161 (CLEC2D–KLRB1) signaling between DNTs and CD56^dim^ NK cells was particularly prominent. Prior studies have shown that this signaling axis can costimulate T cells, enhancing their activation and cytokine secretion while suppressing NK-cell cytotoxicity [35,36]. In addition, human leukocyte antigen (HLA) family ligand–receptor pairs between DNTs and other immune subsets were broadly strengthened, consistent with increased antigen presentation and T cell activation [37]. Together, these findings suggest that DNTs may participate in the dysregulated immune communication network observed in SLE. A more detailed bubble plot illustrating common DNT ligand–receptor interactions is provided in Fig. S8.

Building on the communication-level insights, we investigated the intrinsic molecular regulators of DNTs. Specifically, we integrated DNT cells from both SLE and control samples (Fig. 4H). For each sample, the top 200 SarsGT attention-ranked genes were identified, and cross-sample occurrence frequencies were calculated. Genes with low occurrence frequencies were excluded to improve robustness, and representative SLE- and control-specific genes are shown in Fig. 4I, with the complete results provided in Fig. S9 and Table S12 and S13. Functional enrichment analysis using Enrichr [22] revealed significant enrichment of Viral myocarditis, Antigen processing and presentation, and Epstein–Barr virus (EBV) infection pathways (Fig. 4J, FDR < 0.05). Enrichment of antigen processing and presentation pathways is consistent with enhanced immune activation and loss of immune tolerance [38,39], whereas enrichment of the EBV infection pathway is consistent with prior evidence implicating EBV in SLE pathogenesis [40]. Collectively, integration of pathway enrichment and inferred intercellular communication analyses suggests that DNTs are linked to multiple immune-related pathways and may contribute to the dysregulated immune environment characteristic of SLE.

### Identification of disease-associated regulatory modules and potential targets in the DNT transcription factor network within SLECA

**Fig. 5.**
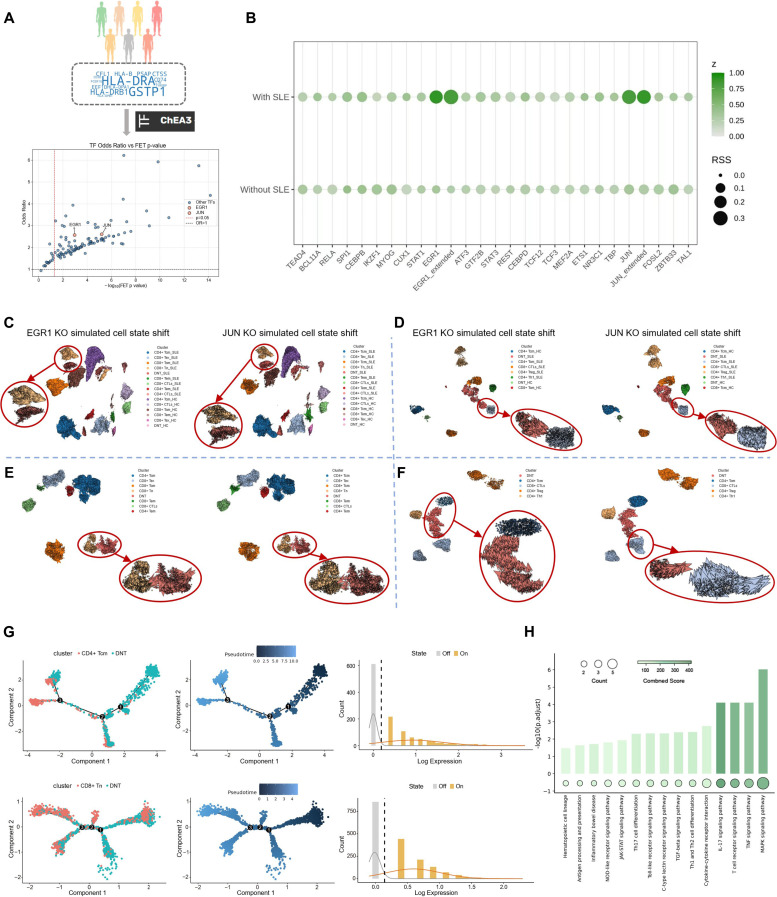
Functional characterization of JUN as a candidate transcription factor in DNT cells. (A) Transcription factor prediction based on high-frequency important genes identified from DNT cells in both with SLE and without SLE groups using ChEA3. The scatter plot displays enrichment results, with the *x* axis representing FET significance (−log_10_ *P* value) and the *y* axis representing enrichment strength (odds ratio). Key transcription factors EGR1 and JUN are highlighted, with the red dashed line indicating the significance threshold (P=0.05) and the gray dashed line indicating an odds ratio (OR) of 1. (B) SCENIC-based regulon activity analysis of ChEA3-enriched candidate transcription factors in DNT cells from SLE and control samples. Dot color indicates the standardized AUCell score, and dot size represents the regulon specificity score (RSS). EGR1- and JUN-related regulons, including EGR1, EGR1_extended, JUN, and JUN_extended, showed higher inferred activity in DNT cells from SLE samples. (C) Simulated cell state shifts in PBMC-derived T cell subsets following EGR1 knockout (left) and JUN knockout (right), based on the combined SLE–control dataset. Colors indicate distinct T cell clusters. Zoomed-in insets are included to improve the visibility of local perturbation vectors and highlight representative regions with notable predicted cell-state changes. (D) Simulated cell state shifts in skin biopsy-derived T cell subsets following EGR1 knockout (left) and JUN knockout (right), based on the combined SLE–control dataset. (E) Simulated cell state shifts in PBMC-derived T cell subsets following EGR1 knockout (left) and JUN knockout (right), based on the SLE-only dataset. (F) Predicted cell state shifts in skin biopsy-derived T cell subsets following simulated EGR1 knockout (left) and JUN knockout (right), based on the SLE-only dataset. (G) Monocle2 pseudotime trajectory analysis of T cell subsets, including DNT cells, following simulated 50% JUN down-regulation, with GeneSwitch identifying JUN as a candidate switch gene associated with the inferred DNT-cell state transition. The dashed vertical line indicates the expression threshold used to distinguish the 2 states. (H) Visualization of enrichment analysis of JUN-associated target genes inferred by SCENIC.

Building on the gene and function level findings, we next probed transcriptional regulation underlying DNT biology. We applied ChEA3 [41] to the high-frequency gene set of DNTs derived from both SLE and control samples to predict upstream transcription factors (Methods). This analysis identified a restricted set of transcription factors significantly associated with the DNT-related genes (Fig. 5A), suggesting the presence of a limited group of candidate upstream regulators. To determine which of these candidate transcription factors exhibited active regulatory programs in DNT cells, we next applied SCENIC [28] to DNT cells from SLE and control samples and assessed their inferred regulon activity patterns (Fig. 5B). The results showed that, among these candidate factors, EGR1 and JUN exhibited prominent regulon activity in SLE-derived DNT cells.

Based on these results, we next performed in silico transcription factor perturbation analysis using CellOracle [25] to evaluate whether EGR1 and JUN may be associated with the maintenance of DNT-cell states. EGR1 has previously been implicated in DNT-cell differentiation and T cell activation [42,43], whereas JUN, a major component of the AP-1 transcription factor complex, is known to regulate T cell activation, effector function, and lineage differentiation [44,45]. In both the combined SLE–control dataset (Fig. 5C and D) and the SLE-only dataset (Fig. 5E and F), CellOracle simulations predicted that inhibition of either JUN or EGR1 would be associated with a shift of DNT cells toward conventional CD4^+^ and CD8^+^ T cell states. To assess whether the perturbation-associated cell-state patterns observed in the integrated analysis were reproducible at the dataset level, we repeated the CellOracle-based perturbation analysis in individual datasets analyzed separately. Similar predicted shifts were observed in adult PBMC-derived T cells from GSE135779, dermis-derived T cells from GSE179633, and adult PBMC-derived T cells from GSE174188 (Figs. S10 to S12), supporting the consistency of the inferred perturbation-associated patterns across different cohort and tissue contexts. Compared with EGR1, the potential role of JUN in maintaining SLE-associated DNT-cell states has not been clearly defined. Therefore, we further examined the relationship between JUN activity and DNT-cell state trajectories. Pseudotime analysis using Monocle2 [26] suggested that simulated 50% down-regulation of JUN expression was associated with a shift in inferred developmental trajectories toward CD4^+^ central memory T (Tcm) and CD8^+^ naive T (Tn) lineages (Fig. 5G). Consistently, GeneSwitch [27] identified JUN as a potential “switch” gene associated with DNT-cell fate transitions. Similar findings were reproduced across additional datasets (Figs. S13 to S17), supporting a potential association between JUN activity and the maintenance of DNT-cell states.

Having established a potential role for JUN in DNT-cell biology, we next examined JUN-centered regulatory programs in SLE. Comparison of JUN regulatory networks between SLE and control samples revealed a substantial expansion in the number of inferred target genes under disease conditions (Fig. S18), suggesting enhanced regulatory connectivity. Several inferred targets, including *NFKBIA*, *TNF*, *FOS*, and *TNFAIP3*, have previously been implicated in SLE susceptibility and pathogenesis [46]. Functional enrichment analysis of JUN-regulated genes further revealed significant enrichment in pathways associated with immune activation and autoimmunity, including TCR signaling, interleukin-17 (IL-17) signaling, mitogen-activated protein kinase (MAPK) signaling, TNF signaling, and antigen processing and presentation (Fig. 5H). Collectively, these results identify JUN as a candidate transcriptional regulator associated with DNT-cell identity and disease-related immune programs in SLE, providing a basis for future mechanistic investigation.

## Discussion

We developed SLECA, an integrated single-cell transcriptomic atlas for SLE that consolidates 8 studies comprising 366 samples. By combining standardized metadata with a graph-transformer-based framework, SLECA enables systematic cross-cohort comparisons across age, sex, tissue source, and disease state. In addition to conventional differential expression analysis, the atlas incorporates attention scores as a model-based and non-causal indicator of gene importance, providing a complementary model-driven perspective for interpreting genes associated with cell-state variation.

A key finding of this study is the enrichment of DNT cells in SLE relative to controls, as reflected by both higher detection frequency and greater relative abundance. Within this population, JUN showed increased regulatory activity and was associated with multiple inflammation- and immunity-related pathways, while in silico perturbation suggested that JUN inhibition may shift DNT cells toward conventional T cell states. Although these findings remain correlational and require experimental validation, SLECA provides a consistent and reusable framework for cross-dataset validation, enabling more robust evaluation of rare populations such as DNT cells and facilitating mechanistic hypothesis generation. More broadly, SLECA addresses 2 major challenges in current SLE single-cell research: fragmented scRNA-seq resources and limited robustness of rare-cell analysis across heterogeneous cohorts.

Several limitations should be noted. The current version of SLECA is based primarily on transcriptomic data and is dominated by PBMC samples, whereas pediatric, male, and tissue-biopsy samples remain underrepresented. Although scVI was used for batch correction, residual batch effects may persist across studies and platforms. In addition, the regulatory associations involving JUN and EGR1 are derived from computational perturbation analyses and GRN inference. While these approaches enable systematic exploration of candidate regulatory programs at single-cell resolution, they remain hypothesis-generating and do not establish direct causal mechanisms. Therefore, experimental validation will be required to confirm the predicted roles of JUN and EGR1 in DNT-cell state transitions and inflammatory transcriptional programs. Such validation may include CRISPR-mediated knockdown or overexpression, perturbation assays, lineage-tracing experiments, protein-level validation, and chromatin accessibility profiling. Future updates will expand cohort diversity and incorporate spatial transcriptomics and multi-omics data to refine the mechanistic and tissue-contextual understanding of DNT cells and other disease-relevant rare populations. Furthermore, integrating single-cell multi-omics profiling with targeted perturbation experiments may facilitate validation of the predicted regulatory relationships and provide deeper insights into the molecular mechanisms underlying DNT-cell dysfunction in SLE. Overall, SLECA provides an open, interpretable, and reusable single-cell resource for the community, accelerating the integration of multi-cohort data into biologically and clinically meaningful hypotheses for SLE research.

## Data Availability

All single-cell datasets used in this paper are publicly available. Data-1 was obtained from GEO under accession number GSE135779. Data-2 was obtained from GEO under accession number GSE142016. Data-3 was obtained from GEO under accession number GSE179633. Data-4 was obtained from GEO under accession number GSE162577. Data-5 was obtained from GEO under accession number GSE186476. Data-6 was obtained from GEO under accession number GSE174188. Data-7 was obtained from GEO under accession number GSE250024. Data-8 was obtained from GEO under accession number GSE158055. The processed SLECA atlas resource is publicly accessible through the SLECA webserver at http://sleca-repository.com/, where users can browse, query, and download processed atlas data. The source code and analysis pipeline are available at https://github.com/Snnrriet/SLECA. The GitHub repository provides the implementation of SarsGT and related analysis scripts.

## Supplementary Materials

Supplementary 1 Figs. S1 to S18 Tables N1 to N4 Tables S1 to S13
