# Supplementary material for: SLECA: A Single-Cell Atlas of Systemic Lupus Erythematosus Enabling Rare-Cell Discovery Using Graph Transformer
Source: Comput Struct Biotechnol J. 2026 Jul 13;35(1):0163. doi: 10.34133/csbj.0163 (PMC13358165; doi:10.34133/csbj.0163)
Supplement: Supplementary 1 — Figs. S1 to S18 Tables N1 to N4 Tables S1 to S13 [file csbj.0163.f1.zip › Supplementary.docx]

*Supplementary Information*

**SLECA: a single-cell atlas of systemic lupus erythematosus enabling rare cell discovery using graph transformer**

**Supplementary Figures**


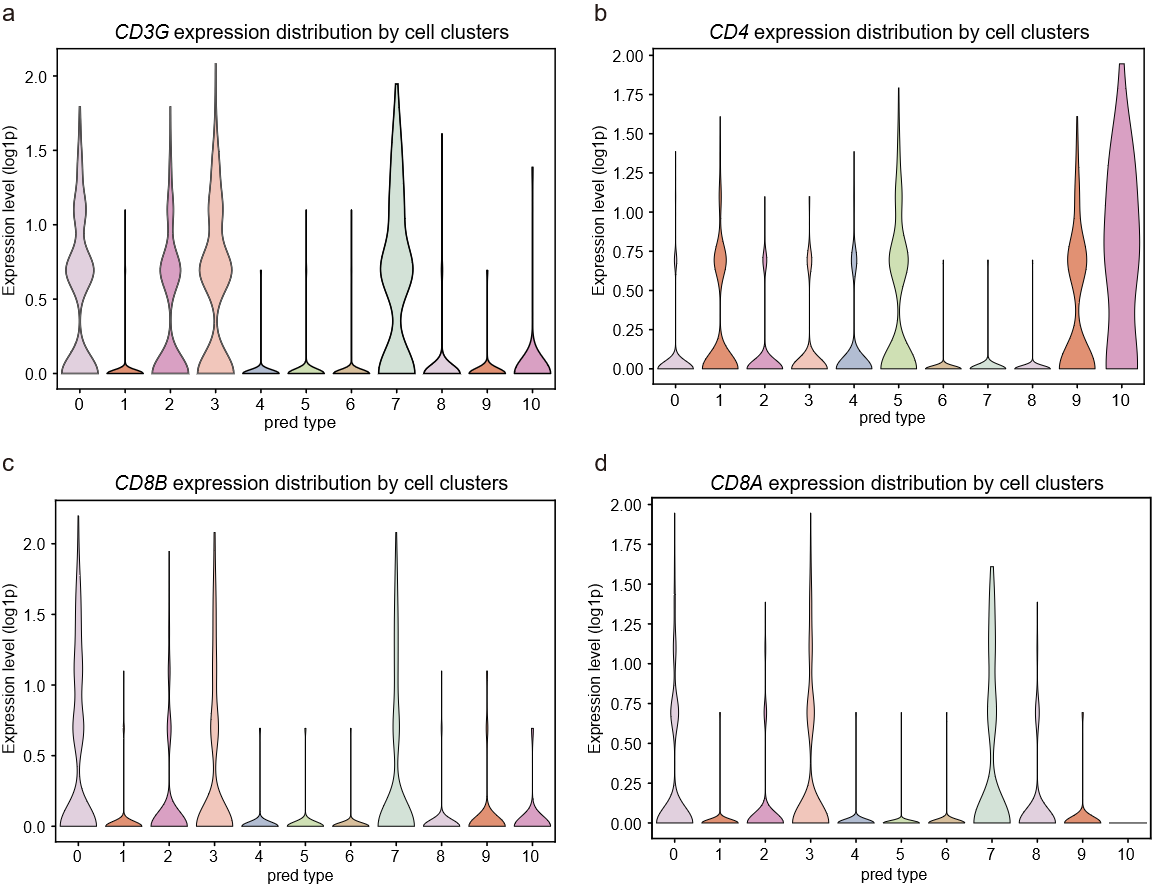


**Supplementary Fig S1.** Violin plots showing the expression of *CD3G*, *CD4*, *CD8B*, and *CD8A* across cell types.


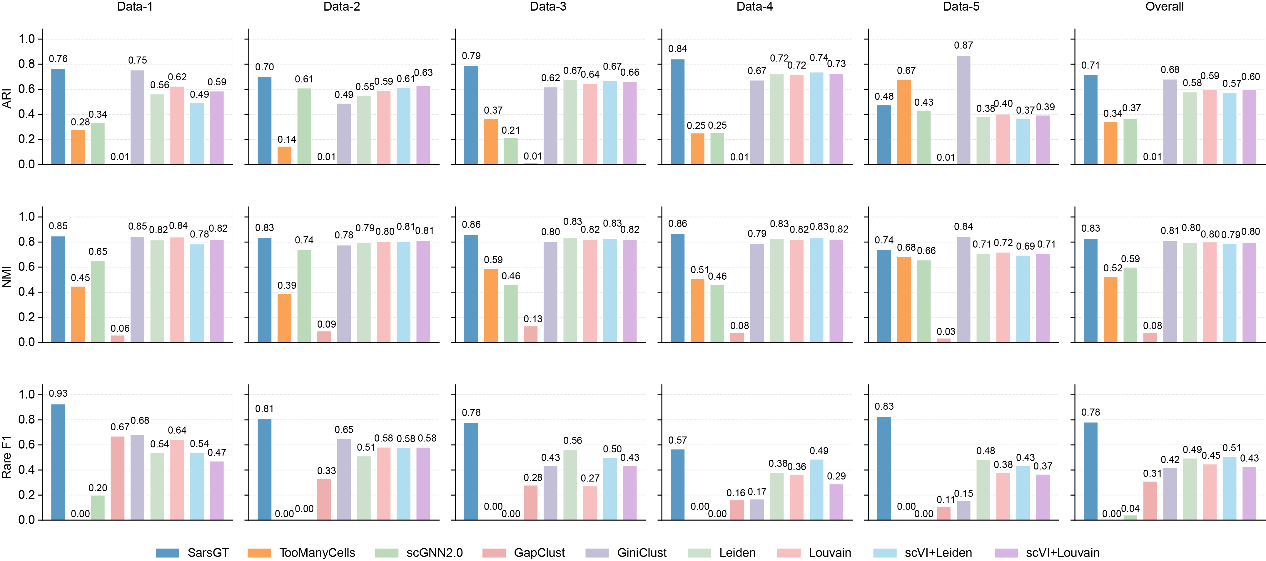


**Supplementary Fig S2. Benchmarking of SarsGT against conventional and rare-cell-oriented clustering methods.** Benchmarking of SarsGT and compared methods across five annotated public scRNA-seq datasets and the overall summary. The compared methods include Leiden, Louvain, scVI+Leiden, scVI+Louvain, GiniClust, TooManyCells, GapClust, scGNN2.0, and SarsGT. Performance was evaluated using adjusted Rand index (ARI), normalized mutual information (NMI), and rare-cell F1 score. Rare cell types were defined as cell types accounting for less than 3% of the total cells in each dataset. Bar labels indicate metric values. The overall panel summarizes performance across the five benchmark datasets.


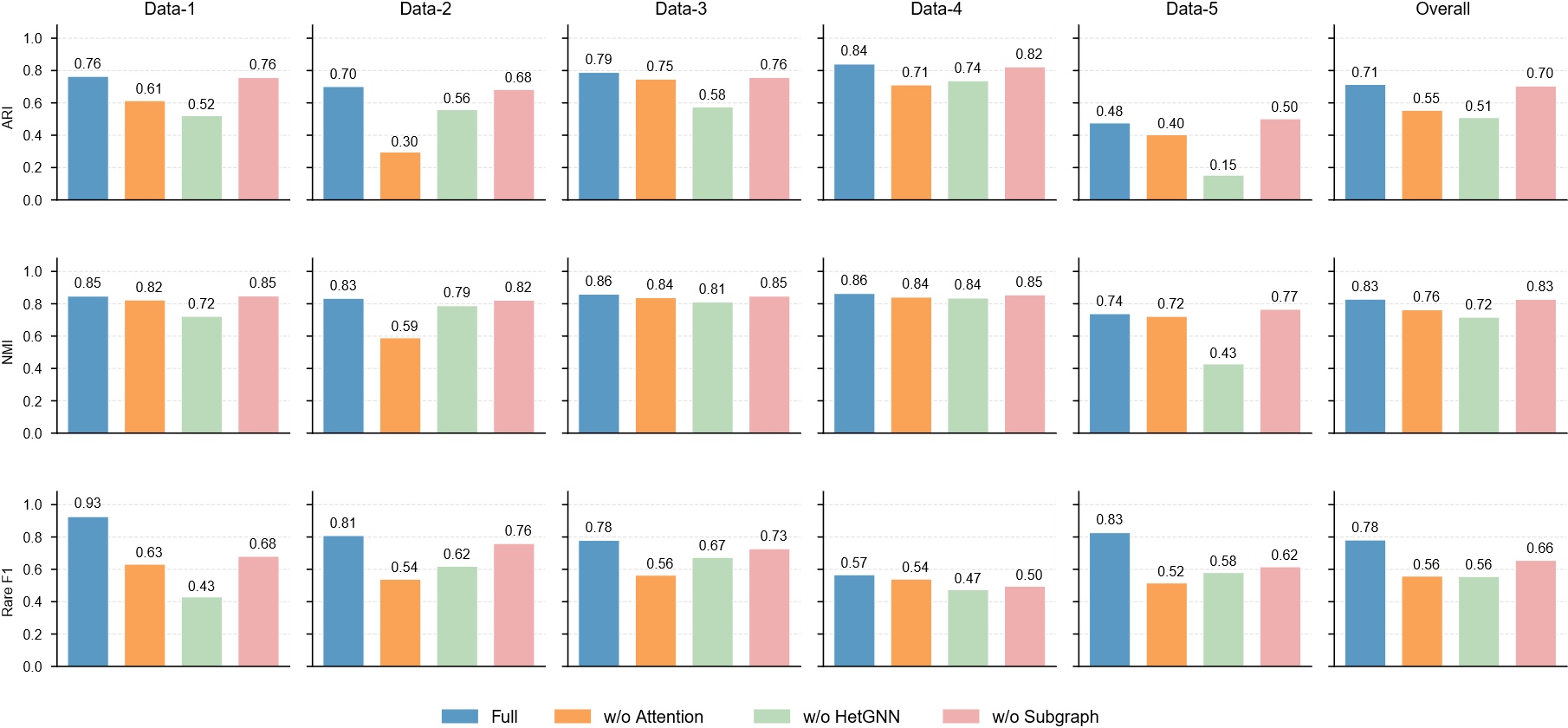


**Supplementary Fig S3. Ablation analysis of key SarsGT components.** Ablation analysis of SarsGT across five annotated public scRNA-seq benchmark datasets and the overall summary. The full SarsGT model was compared with three ablated variants: removal of the attention mechanism, removal of the heterogeneous graph structure, and removal of the subgraph sampling strategy. Performance was evaluated using adjusted Rand index (ARI), normalized mutual information (NMI), and rare-cell F1 score. Bar labels indicate metric values. The overall panel summarizes performance across the five benchmark datasets.

**
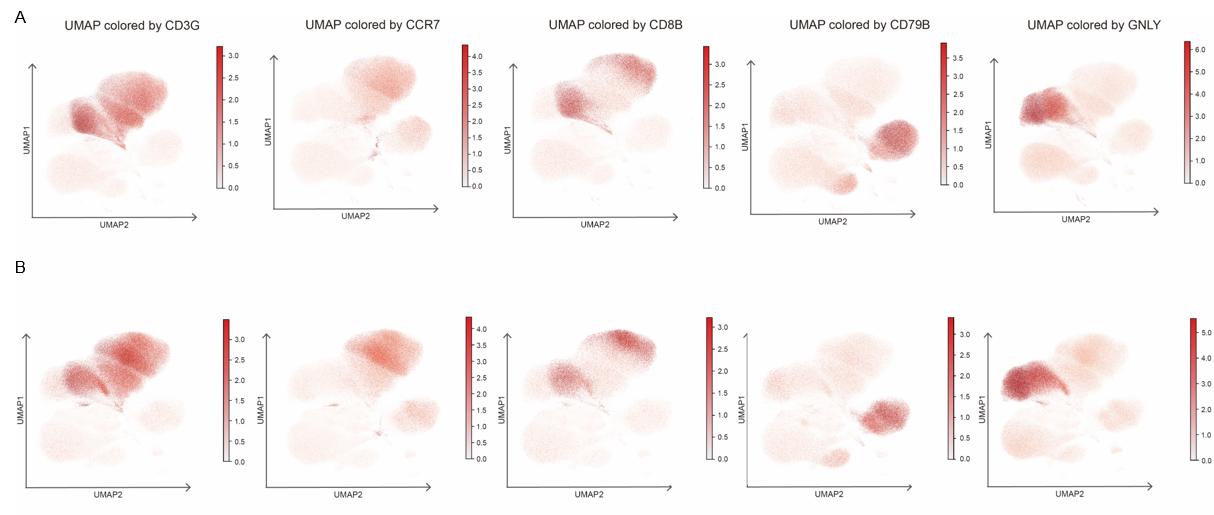
**

**Supplementary Fig S4.** **A)** Displays marker gene expression for major cell types in the within SLE group, **B)** Displays marker gene expression for major cell types in the without SLE.


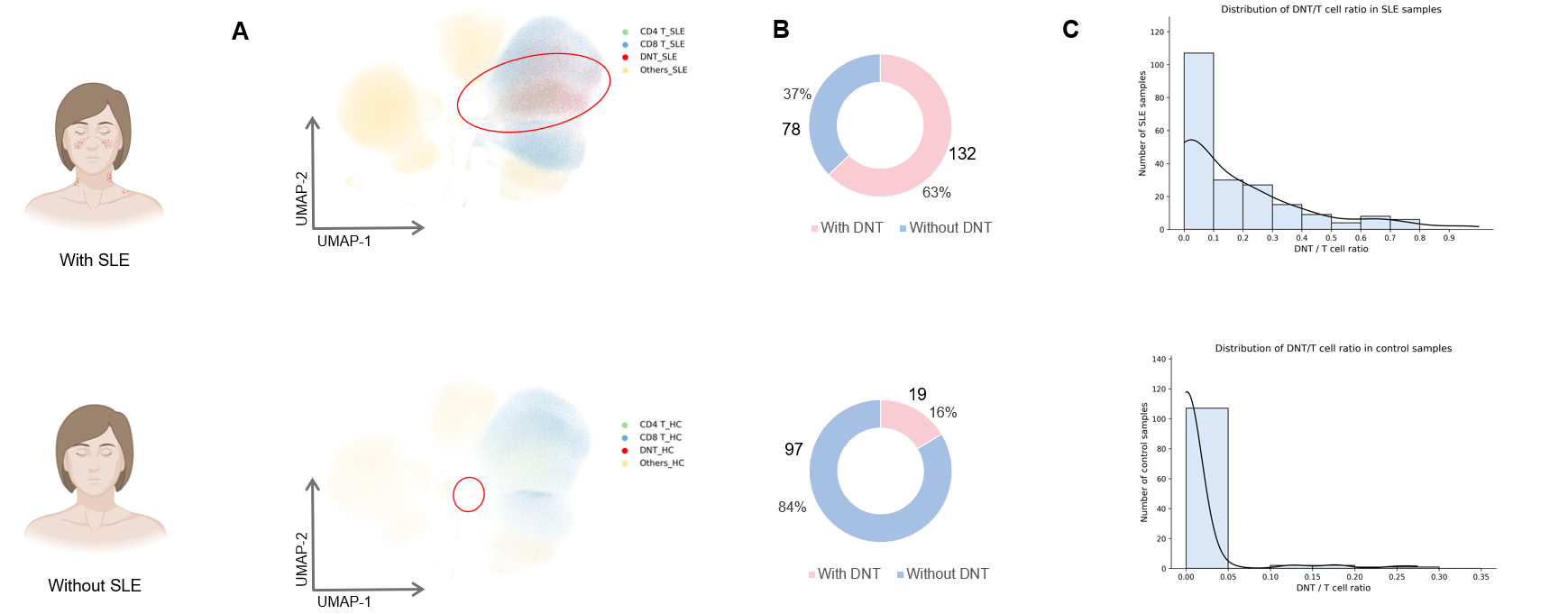


**Supplementary Fig S5. PBMC-only sensitivity analysis of DNT-cell enrichment in SLE.** **A)** UMAP visualization of T-cell subsets in SLE and healthy control samples, with DNT-cell regions highlighted. **B)** Sample-level proportion of SLE and healthy control samples with or without detectable DNT cells. DNT cells were detected in 63% of SLE samples and 16% of healthy control samples. **C)** Distribution of sample-level DNT/T cell ratios in SLE and healthy control samples. The PBMC-only analysis shows that DNT cells remain enriched in SLE samples compared with healthy controls after excluding skin-derived samples and unmatched external controls.


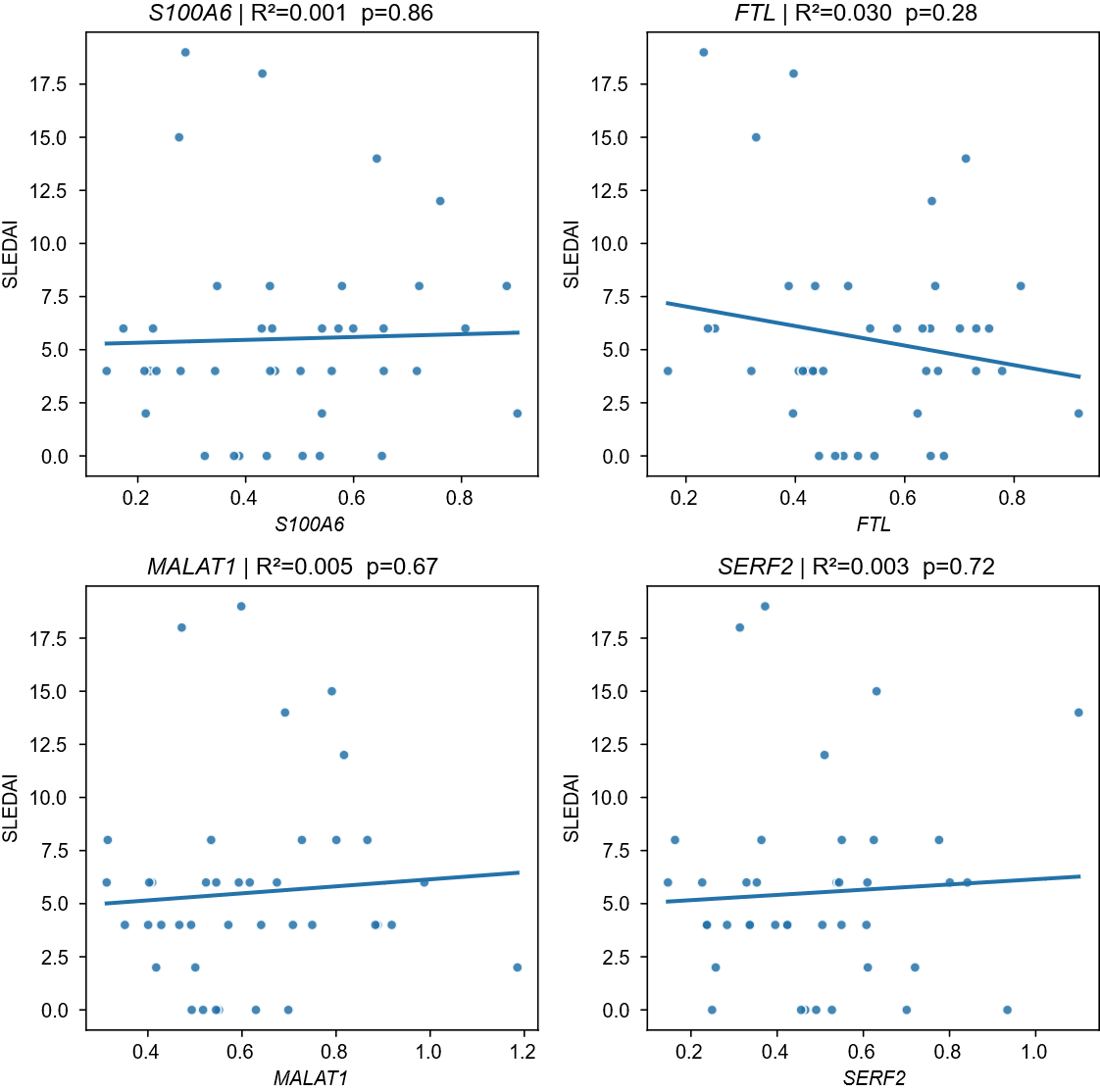


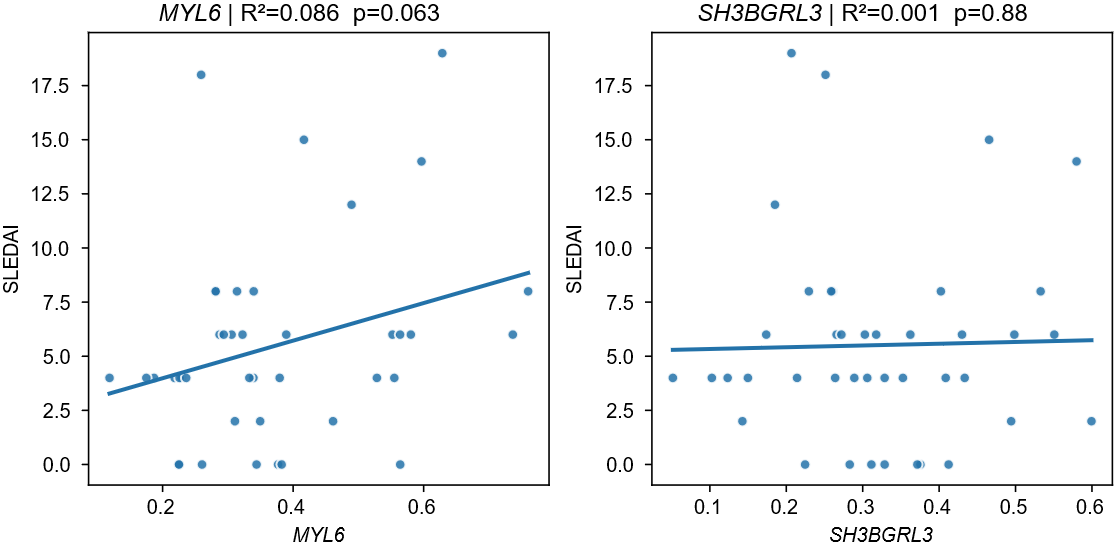


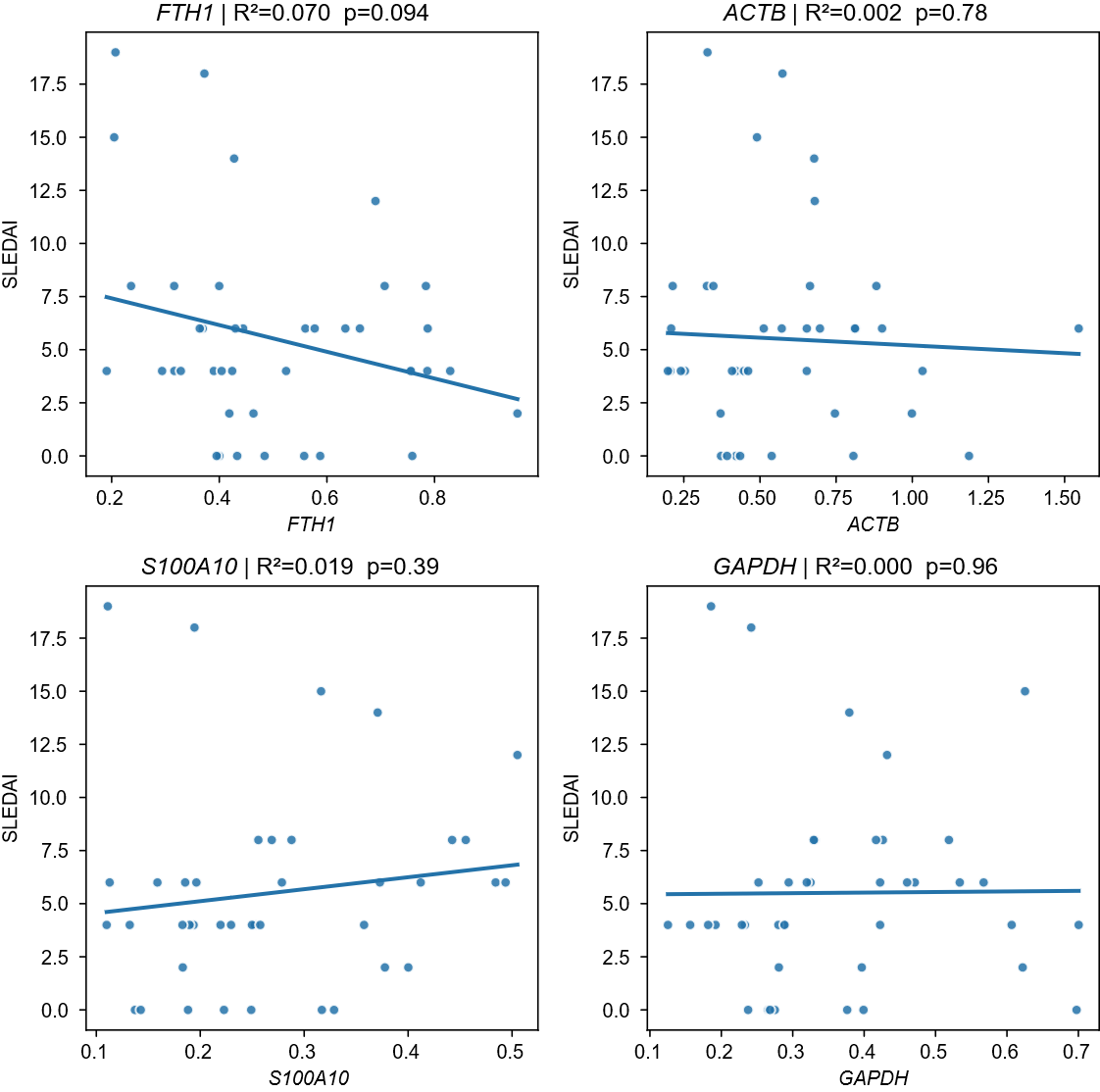


**Supplementary Fig S6.** We selected the top 10 genes with the highest attention scores (*S100A6*, *FTL*, *FTH1*, *ACTB*, *MYL6*, *MALAT1*, *SERF2*, *S100A10*, *GAPDH*, and *SH3BGRL3*) and constructed multiple linear regression models to quantify their associations with disease activity (SLEDAI). Each panel depicts the relationship between the mean attention value of a single gene and the SLEDAI score across SLE samples, where blue dots represent individual samples and the red line indicates the fitted linear regression.


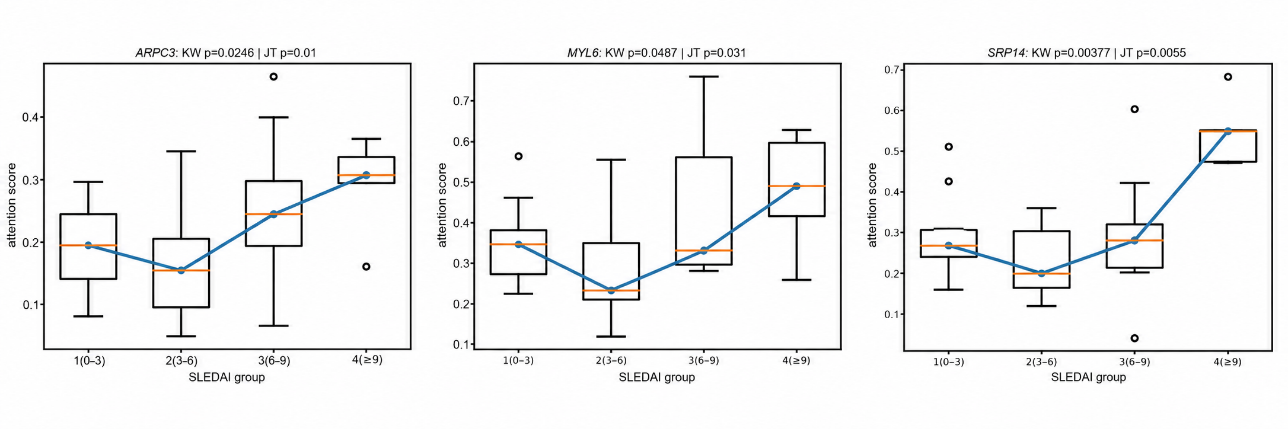


**Supplementary Fig S7.** *ARPC3*, *MYL6*, *SRP14* attention scores across four SLEDAI strata (0-3, 3-6, 6-9, ≥9), showing a significant upward trend with disease activity.


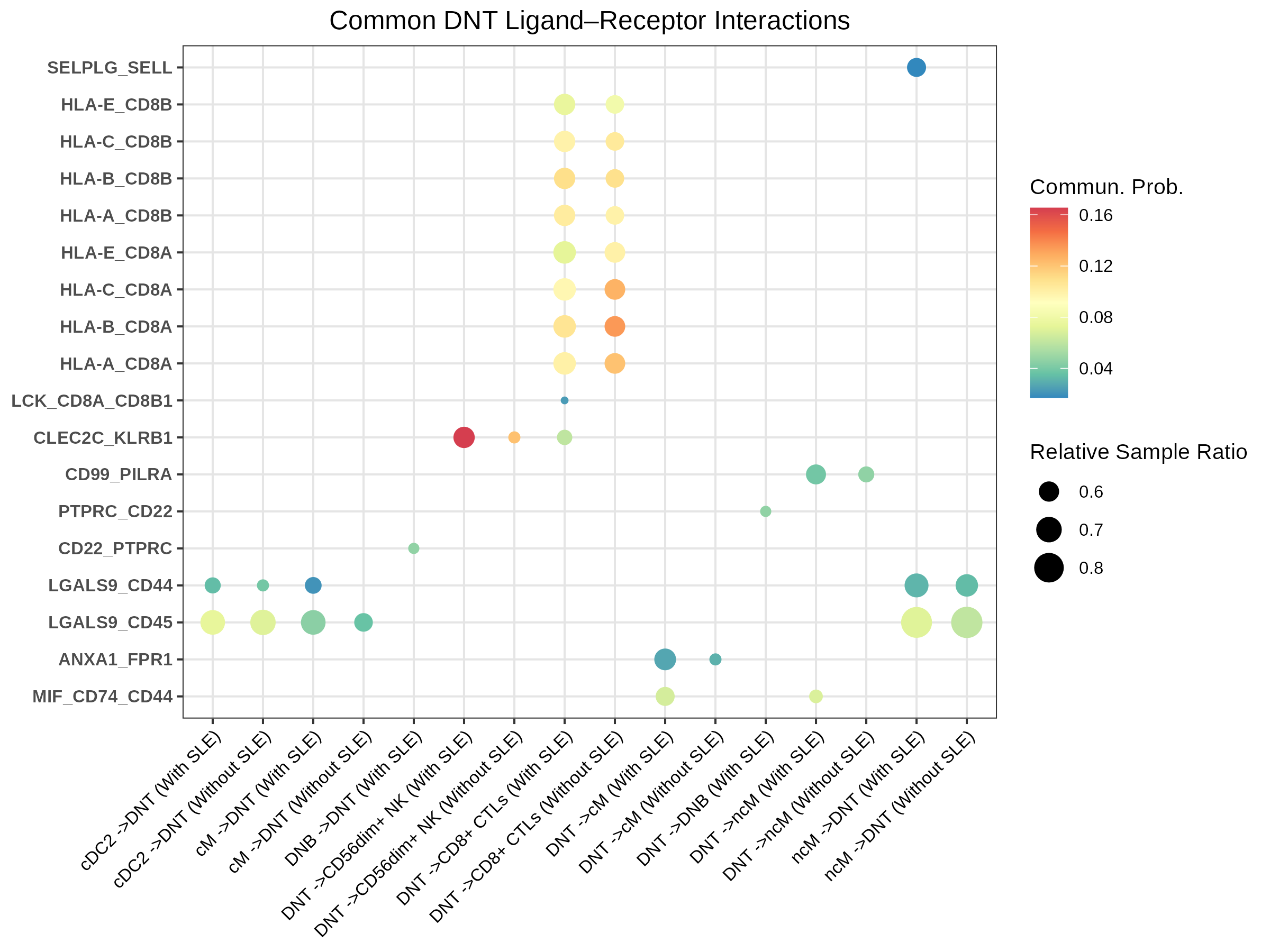


**Supplementary Fig S8.** Cell–cell communication networks inferred by CellChat in SLE and control groups.


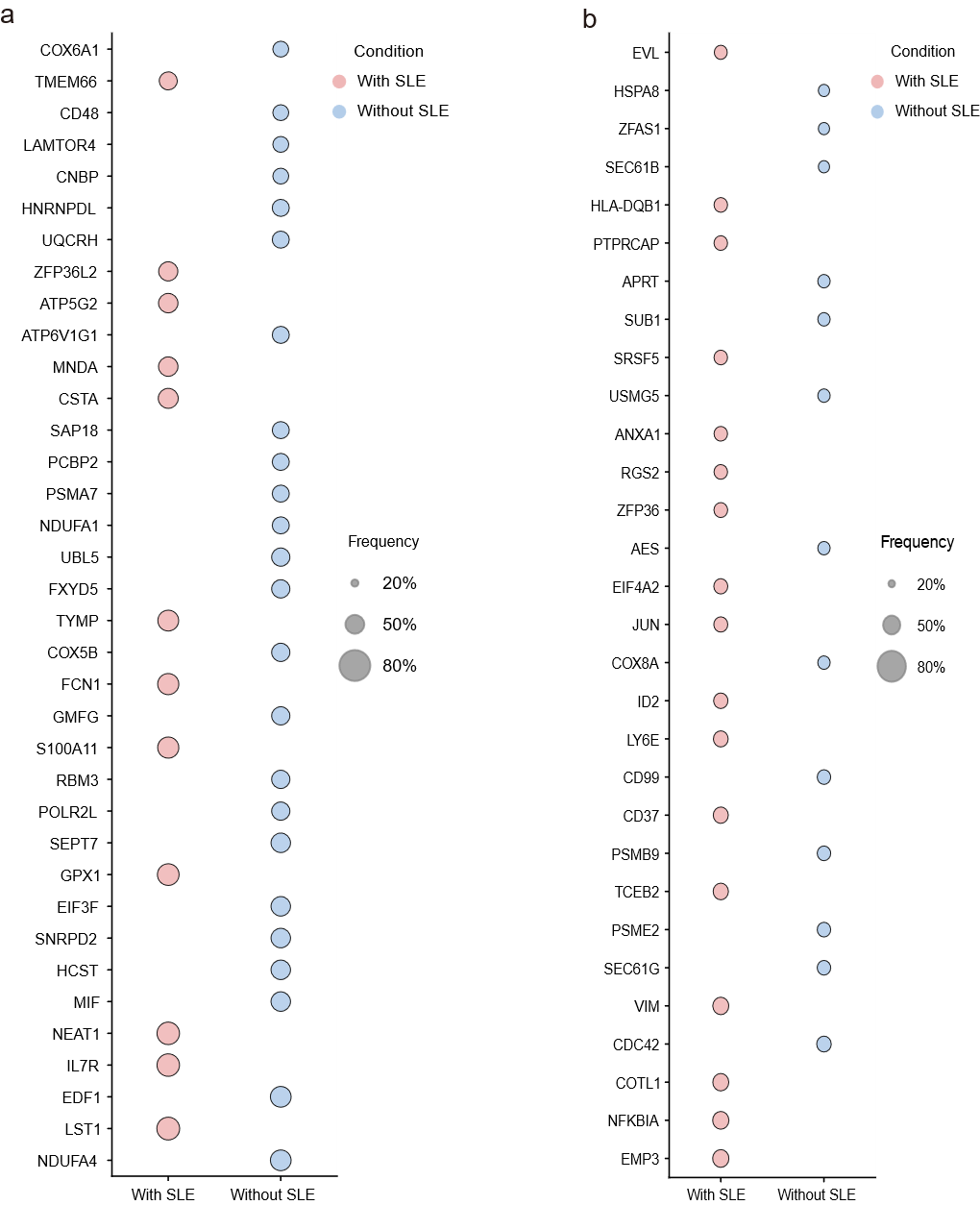


**Supplementary Fig S9.** Bubble plot showing the distribution and occurrence frequencies of group-specific genes among the top 150 genes in SLE and control DNT cells.


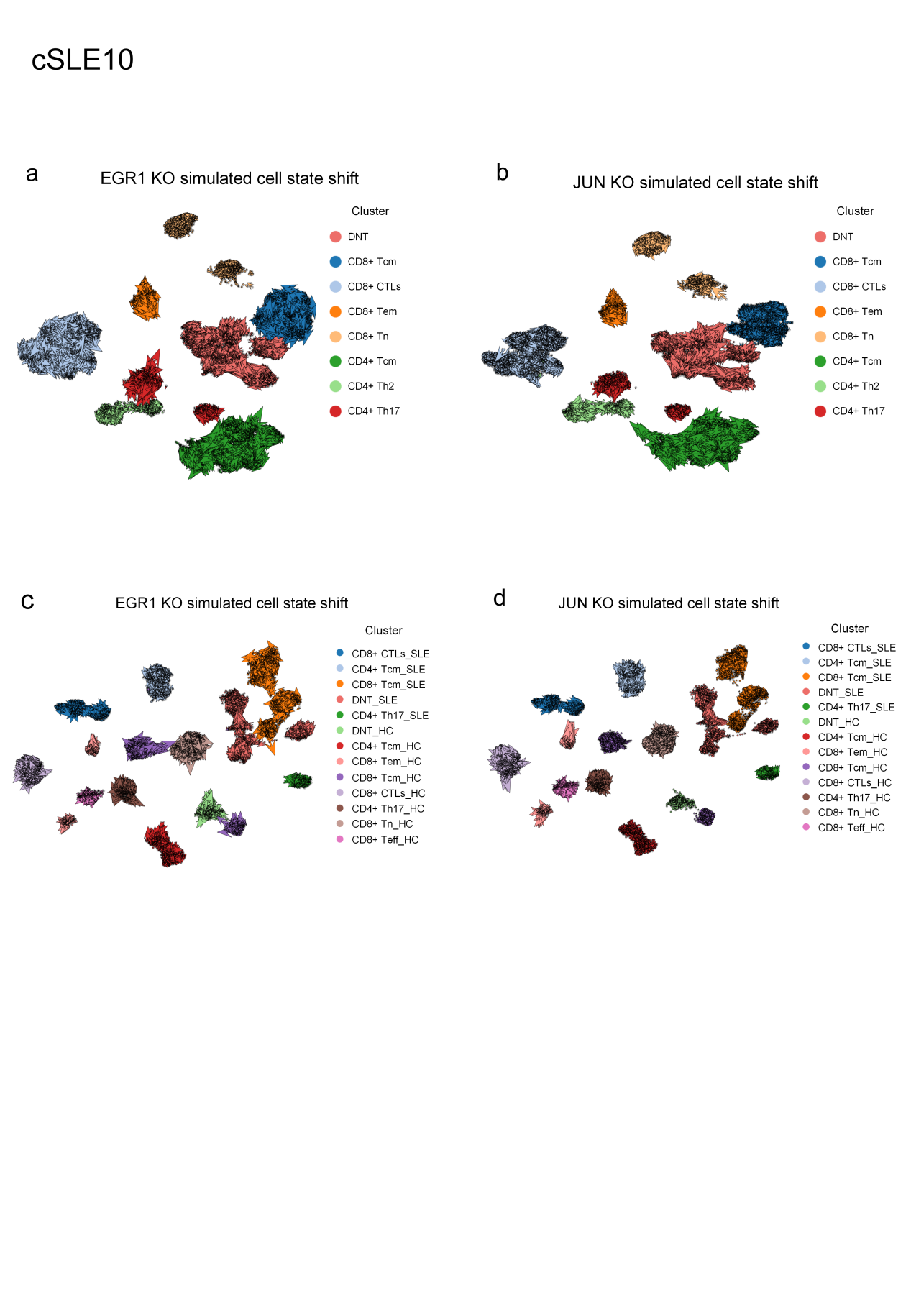


**Supplementary Fig S10.** Extrapolated future states (arrows) after *EGR1* or *JUN* knockout in PBMC-derived T cell subsets from adult samples (GSE135779)^[^[^1^](#_ENREF_1)^]^. **a**, **b**: Predicted cell-state trajectories following *EGR1* and *JUN* knockout, respectively, based on SLE samples only. **c**, **d**: Corresponding extrapolated future states after *EGR1* and *JUN* knockout integrating both SLE and healthy control samples. Colors indicate distinct T cell clusters.


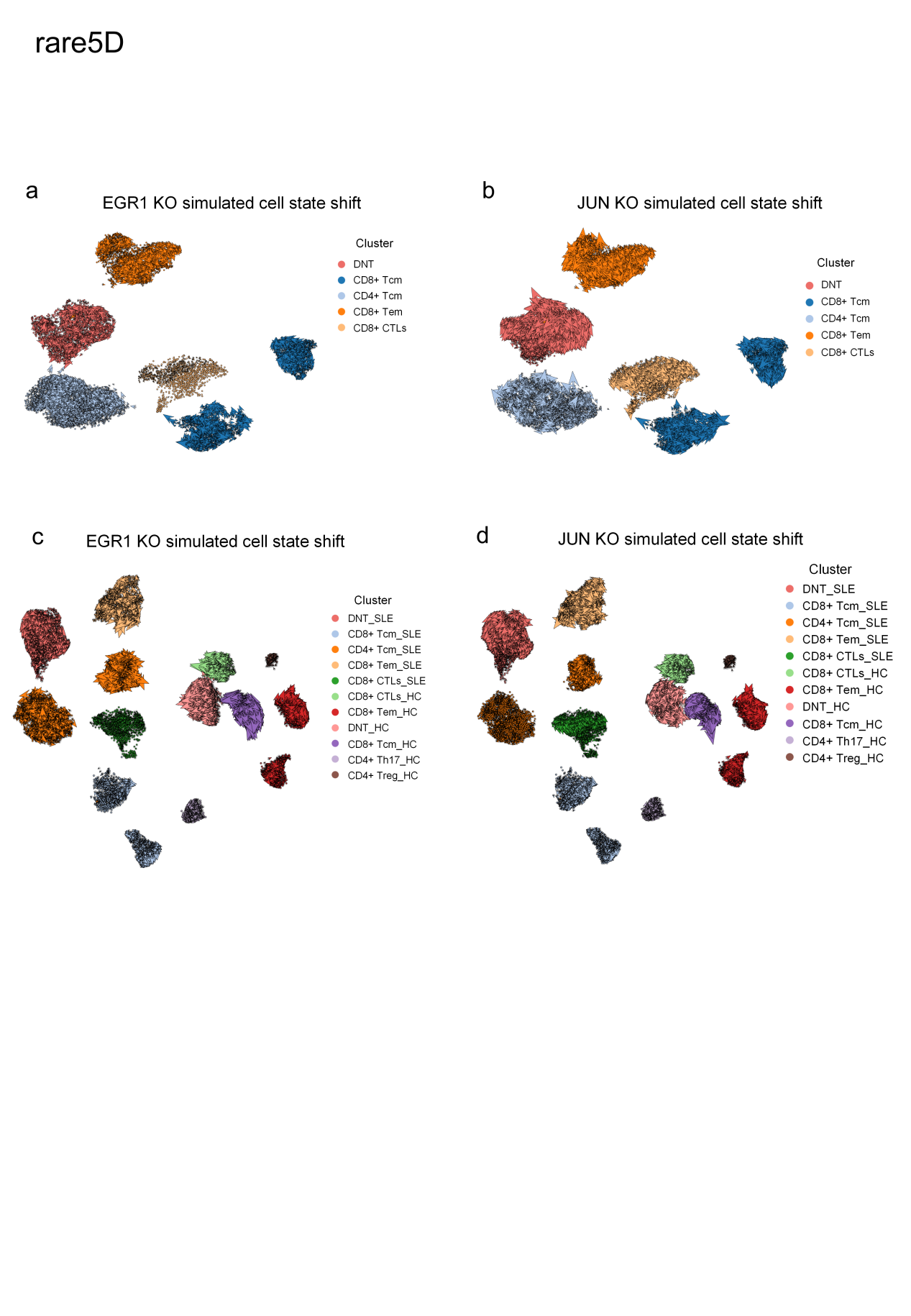


**Supplementary Fig S11.** Extrapolated future states (arrows) after *EGR1* or *JUN* knockout in dermis-derived T cell subsets from skin biopsy samples (GSE179633)^[^[^2^](#_ENREF_2)^]^. **a**, **b**: Predicted cell-state trajectories following *EGR1* and *JUN* knockout, respectively, based on SLE samples only. **c**, **d**: Corresponding extrapolated future states after *EGR1* and *JUN* knockout integrating both SLE and healthy control samples. Colors indicate distinct T cell clusters.


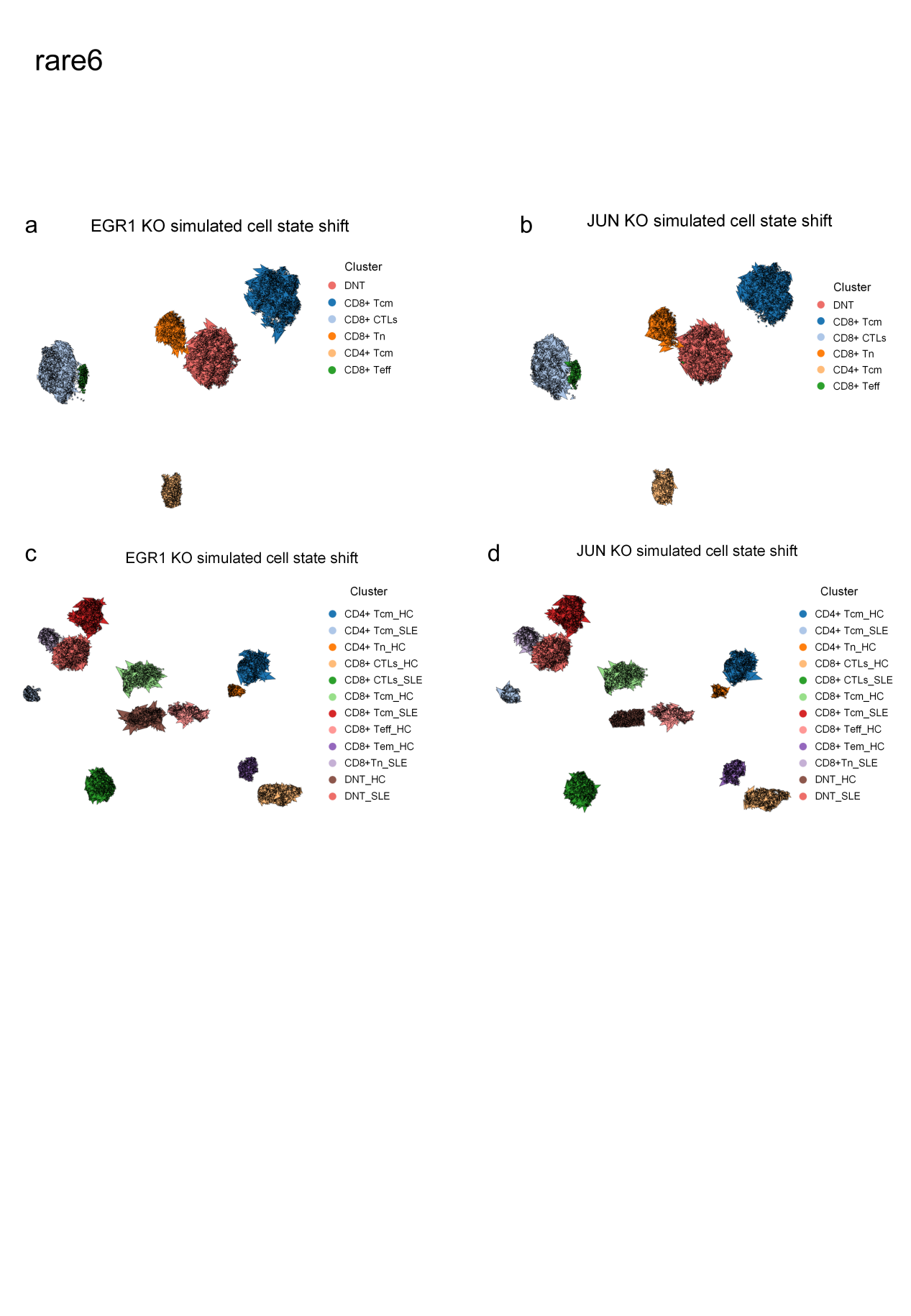


**Supplementary Fig S12.** Extrapolated future states (arrows) after *EGR1* or *JUN* knockout in PBMC-derived T cell subsets from adult samples (GSE174188)^[^[^3^](#_ENREF_3)^]^. **a**, **b**: Predicted cell-state trajectories following *EGR1* and *JUN* knockout, respectively, based on SLE samples only. **c**, **d**: Corresponding extrapolated future states after *EGR1* and *JUN* knockout integrating both SLE and healthy control samples. Colors indicate distinct T cell clusters.

**
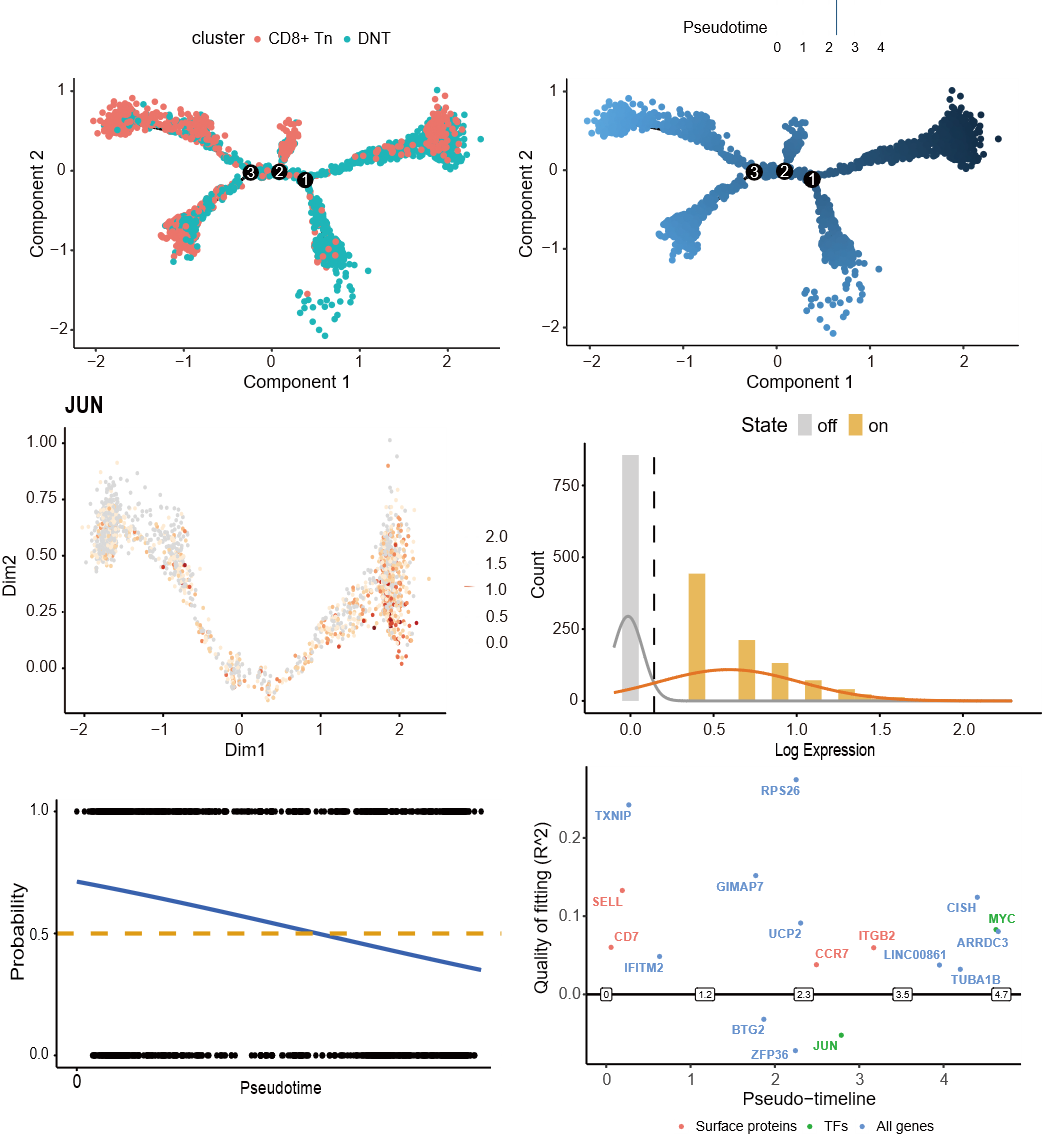
**

**Supplementary Fig S13.** Pseudotime trajectory of T-cell subsets including DNT cells from the pediatric subset of the PBMC dataset GSE135779 inferred with Monocle2, with visualization of the switch gene JUN using GeneSwitch.

**
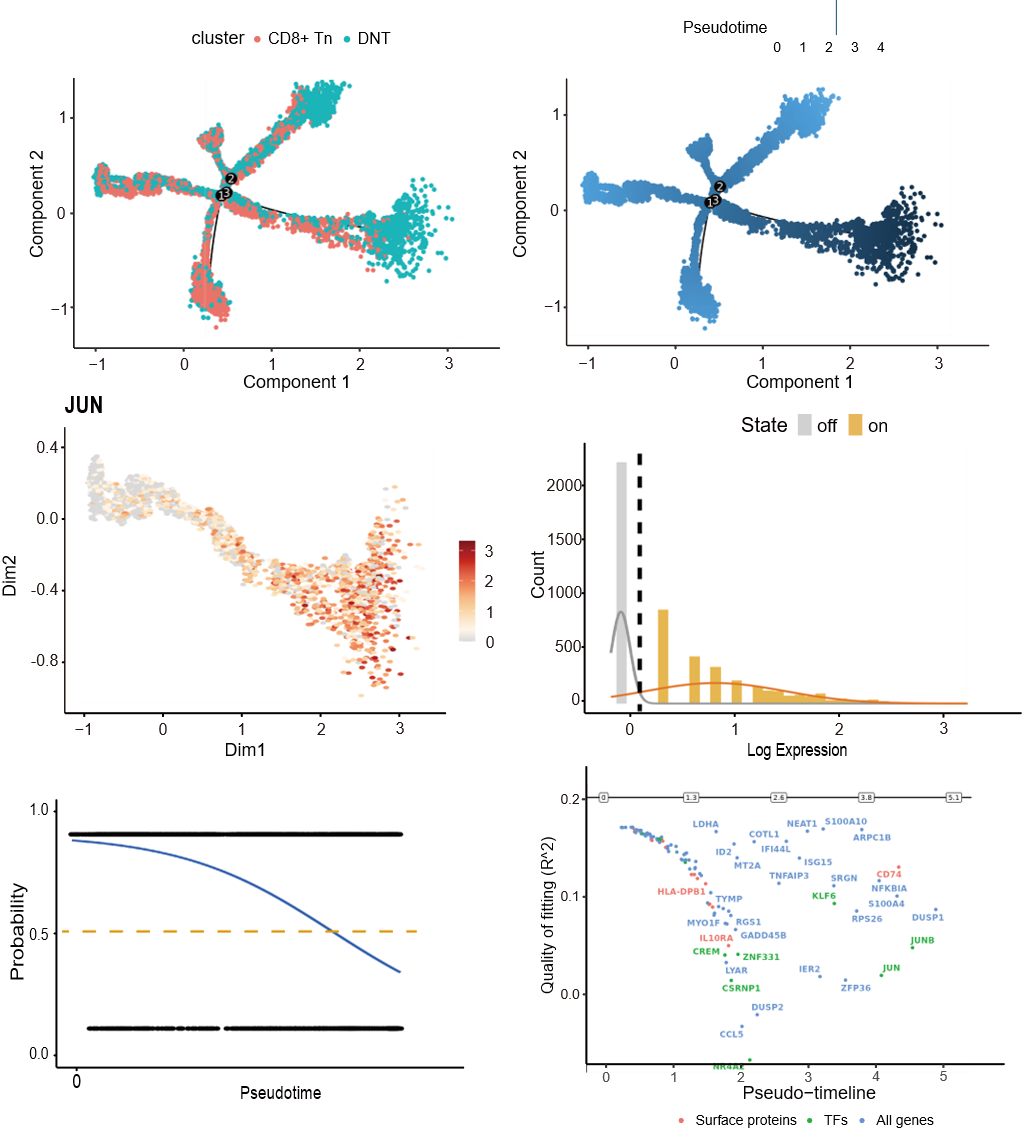
**

**Supplementary Fig S14.** Pseudotime trajectory of T-cell subsets including DNT cells from the adult subset of the PBMC dataset GSE135779 inferred with Monocle2, with visualization of the switch gene JUN using GeneSwitch.


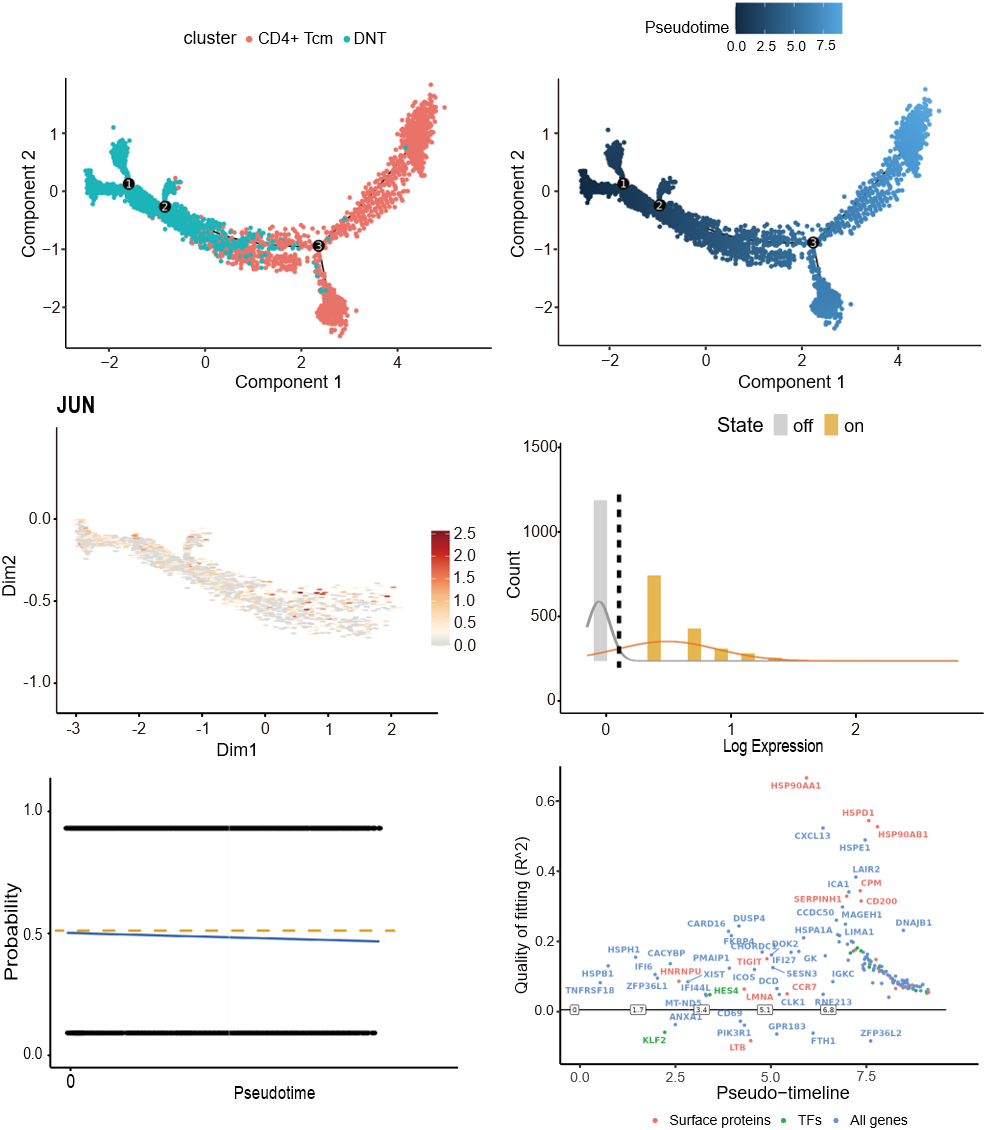


**Supplementary Fig S15.** Pseudotime trajectory of T-cell subsets including DNT cells from the dermal subset of the skin biopsy dataset GSE179633 inferred with Monocle2, with visualization of the switch gene JUN using GeneSwitch.


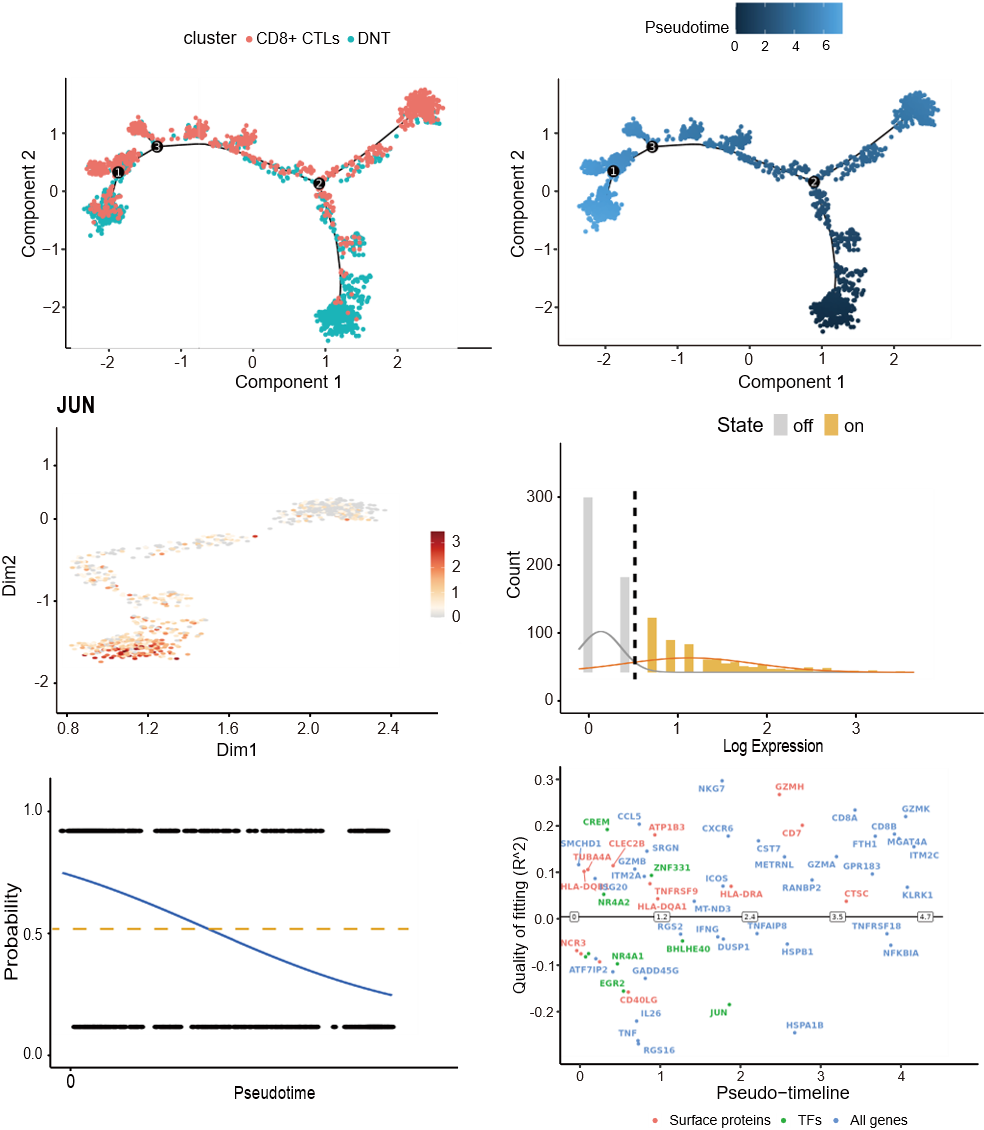


**Supplementary Fig S16.** Pseudotime trajectory of T-cell subsets including DNT cells from the epidermal subset of the skin biopsy dataset GSE179633 inferred with Monocle2, with visualization of the switch gene JUN using GeneSwitch.


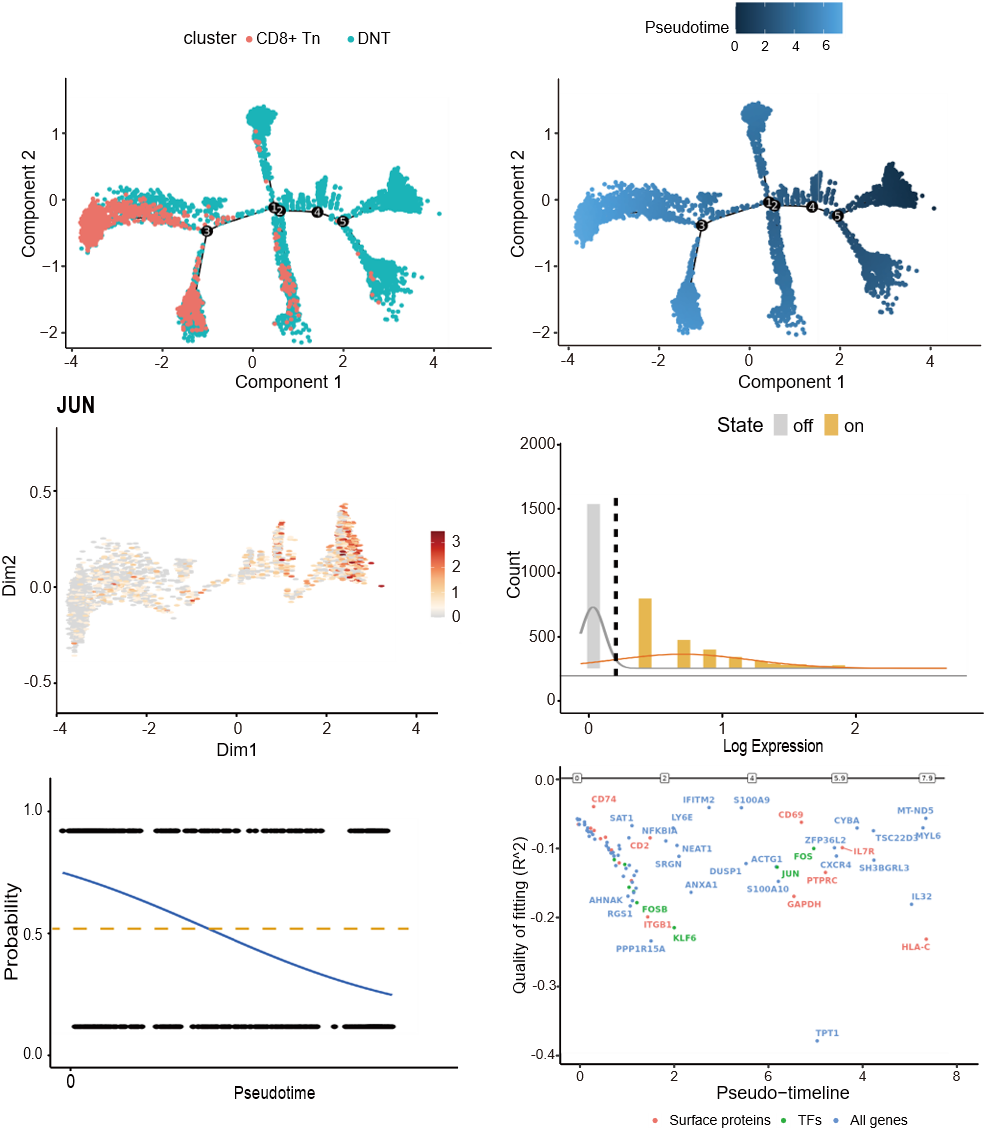


**Supplementary Fig S17.** Pseudotime trajectory of DNT cells from the PBMC dataset GSE174188 inferred with Monocle2, with visualization of the switch gene JUN using GeneSwitch.


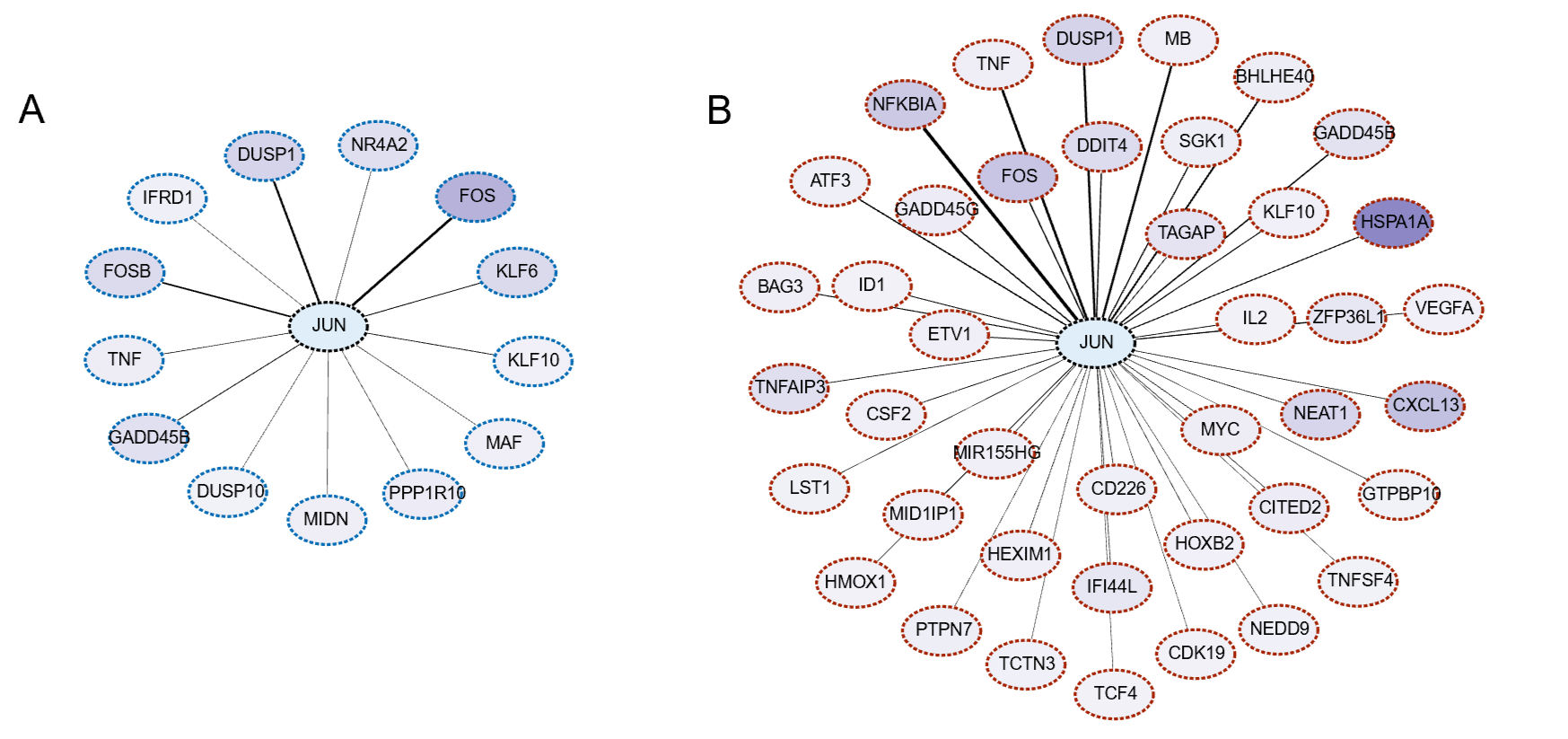


**Supplementary Fig S18. A)** Regulatory network inferred by SCENIC based on predicted JUN-associated target genes in samples without SLE. Nodes represent JUN and its predicted target genes, and edges indicate inferred regulatory associations. **B)** Regulatory network inferred by SCENIC based on predicted JUN-associated target genes in samples with SLE. Compared with the without-SLE network, the with-SLE network shows a denser inferred regulatory structure and stronger associations with several immune-related target genes, including *NFKBIA*, *TNF*, *FOS*, and *TNFAIP3*.

# Supplementary Table

**N1** **Summary of scRNA-seq datasets used for benchmarking**

| Dataset | Context | Species | Cells / Cell types | Accession |
| --- | --- | --- | --- | --- |
| Data-1 | PBMC | Human | 9432 / 13 | 10x Genomics PBMC dataset |
| Data-2 | Liver | Human | 8444 / 20 | GSE115469 |
| Data-3 | Lung (Control) | Human | 6098 / 40 | GSM5226574 |
| Data-4 | Lung (COVID-19) | Human | 3060 / 41 | GSM5226586 |
| Data-5 | Pancreas | Human | 8569 / 14 | GSE84133 |

**N2 Marker gene**

| Cell Type | Marker |
| --- | --- |
| CD4+ Tcm | CD4, CCR7, CD27, CD45RO |
| CD4+ Tn | CD4, CCR7, CD45RA |
| CD4+ Tem | CD45RO, KLRD1, CD27 |
| CD4+ Th1 | CD4, CXCR3 |
| CD4+ Th2 | CD4, GATA3, IL-4 |
| CD4+ Th17 | CD4, IL17A, RORC |
| CD8+ CTLs | CD8A, CD8B, KLRD1, FCGR3A, GNLY |
| CD8+ Tn | CD8A, CD8B, CCR7, CD45RA |
| CD8+ Tcm | CD8A, CD8B, CCR7, CD27, CD45RO |
| CD8+ Tem | CD8A, CD8B, CD45RO, KLRD1, CD27, GNLY, FCGR3A |
| CD8+ Teff | CD8A, CD8B, GZMA, GNLY, FCGR3A |
| DNT | CD3D, CD3E, CD4⁻, CD8A⁻, CD8B⁻ |
| DP T | CD3D,CD3E,CD4,CD8A,CD8B |
| B cells | CD19, CD79A, MS4A1 |
| Activated B Cells | CD19, MS4A1, CD69, HLA-DRA, CD80, TNFRSF13B |
| DNB | CD19, MS4A1, IgD⁻, CD27⁻ |
| Memory B Cells | CD19, MS4A1, CD27, TNFRSF13B, CD80, CD38^-^ |
| Naive B Cells | CD19, MS4A1, IgD, CD27⁻, TCL1A |
| CD56dim+ NK | NCAM1^-^, KLRD1, FCGR3A |
| Macrophages | CD68, CSF1R, CD163 |
| pDCs | IL3RA, LILRA4, TLR7 |
| Plasma Cells | MZB1, CD27, CD38 |
| Plasmablasts | CD27, CD38, MZB1, XBP1, PRDM1, IGHG |
| Monocytes | CD14, FCGR3A, ITGAM |
| Mast cells | CPA3, FCER1A, TPSAB1, TPSB2 |
| Erythroid Cells | HBB, HBA1, HBA2,ALAS2, EPB42 |
| Platelet | ITGA2B, ITGB3 |
| cDC1 | HLA-DRA, CLEC9A, XCR1 |
| cDC2 | CD1C, CLEC10A, ITGAX |
| Megakaryocytes | ITGA2B, MPL, PF4 |
| Neutrophils | FCGR3B, CXCR2 |
| Melanocytes | PMEL, MLANA |
| Fibroblasts | COL1A1, COL3A1 |
| Endothelial | CDH5, VWF |
| Schwann cells | CDH19, MPZ |
| Epithelial | KRT8, KRT18, EPCAM |
| Keratinocytes | KRT5, KRT14 |
| SMC | ACTA2, MYH11 |

**N3 Cell-type proportions in SLE**

| Cell type | Proportion(%) | Cell type | Proportion(%) |
| --- | --- | --- | --- |
| CD4+ Tfh | 0.062494 | Megakaryocytes | 0.022350 |
| cDC/Macro | 0.059571 | CD8+ Temra | 0.019341 |
| CD8+ MAIT | 0.043497 | CD4+ T | 0.010745 |
| Plasmablasts | 0.036276 | CD8+ T | 0.000860 |
| CD4+ Th1 | 0.031978 | Erythroid Progenitors | 0.000774 |
| CD8+ Trm | 0.031032 | Pre-B cells | 0.000774 |
| intMono | 0.025960 | CD4+ Teff | 0.000602 |

**N4 Cell-type proportions in without SLE**

| Cell type | Proportion(%) | Cell type | Proportion(%) |
| --- | --- | --- | --- |
| Megakaryocytes | 0.055083 | CD8+ MAIT | 0.028822 |
| CD8+ Trm | 0.046244 | Platelet | 0.019471 |
| cDC1 | 0.041504 | CD4+ Tregs | 0.018318 |
| CD4+ Treg | 0.041248 | Macro/cDC2 | 0.014475 |
| CD8+ Tex | 0.039199 | Neutrophils | 0.014347 |
| Erythroid cells | 0.012041 |  |  |

**References**

1. Nehar-Belaid, D., et al., *Mapping systemic lupus erythematosus heterogeneity at the single-cell level.* Nature immunology, 2020. **21**(9): p. 1094-1106.

2. Zheng, M., et al., *Single-cell sequencing shows cellular heterogeneity of cutaneous lesions in lupus erythematosus.* Nature communications, 2022. **13**(1): p. 7489.

3. Perez, R.K., et al., *Single-cell RNA-seq reveals cell type–specific molecular and genetic associations to lupus.* Science, 2022. **376**(6589): p. eabf1970.
